# Batf2 differentially regulates tissue immunopathology in Type 1 and Type 2 diseases

**DOI:** 10.1038/s41385-018-0108-2

**Published:** 2018-12-12

**Authors:** Reto Guler, Thabo Mpotje, Mumin Ozturk, Justin K. Nono, Suraj P. Parihar, Julius Ebua Chia, Nada Abdel Aziz, Lerato Hlaka, Santosh Kumar, Sugata Roy, Adam Penn-Nicholson, Willem A. Hanekom, Daniel E. Zak, Thomas J. Scriba, Harukazu Suzuki, Frank Brombacher

**Affiliations:** 1 International Centre for Genetic Engineering and Biotechnology (ICGEB), Cape Town Component, Cape Town, 7925 South Africa; 2 Department of Pathology, University of Cape Town, Institute of Infectious Diseases and Molecular Medicine (IDM), Division of Immunology and South African Medical Research Council (SAMRC) Immunology of Infectious Diseases, Faculty of Health Sciences, University of Cape Town, Cape Town, 7925 South Africa; 3 Wellcome Centre for Infectious Diseases Research in Africa (CIDRI-Africa), Institute of Infectious Diseases and Molecular Medicine (IDM), Faculty of Health Sciences, University of Cape Town, Cape Town, 7925 South Africa; 4 Division of Medical Microbiology, Department of Pathology, Faculty of Health Sciences, University of Cape Town, Cape Town, 7925 South Africa; 5 The Medical Research Centre, Institute of Medical Research and Medicinal Plant Studies (IMPM), Ministry of Scientific Research and Innovation, Yaoundé, Cameroon; 6 Department of Chemistry, Faculty of Science, Cairo University, Cairo, Egypt; 7 RIKEN Center for Integrative Medical Sciences, 1-7-22 Suehiro-cho, Tsurumi-ku, Yokohama, 230-0045 Japan; 8 South African Tuberculosis Vaccine Initiative, Division of Immunology, Department of Pathology, Institute of Infectious Disease and Molecular Medicine, University of Cape Town, Cape Town, 7925 South Africa; 9 The Center for Infectious Disease Research, Seattle, WA 98109 USA

## Abstract

Basic leucine zipper transcription factor 2 (Batf2) activation is detrimental in Type 1-controlled infectious diseases, demonstrated during infection with *Mycobacterium tuberculosis* (Mtb) and *Listeria monocytogenes* Lm. In *Batf2*-deficient mice (Batf2^*−/*−^), infected with Mtb or Lm, mice survived and displayed reduced tissue pathology compared to infected control mice. Indeed, pulmonary inflammatory macrophage recruitment, pro-inflammatory cytokines and immune effectors were also decreased during tuberculosis. This explains that *batf2* mRNA predictive early biomarker found in active TB patients is increased in peripheral blood. Similarly, Lm infection in human macrophages and mouse spleen and liver also increased Batf2 expression. In striking contrast, Type 2-controlled schistosomiasis exacerbates during infected Batf2^*−/*−^ mice with increased intestinal fibro-granulomatous inflammation, pro-fibrotic immune cells, and elevated cytokine production leading to wasting disease and early death. Together, these data strongly indicate that Batf2 differentially regulates Type 1 and Type 2 immunity in infectious diseases.

## Introduction

Batf2 is a transcription factor that belongs to the basic leucine zipper transcription factor family, which includes Batf and Batf3.^1,2^ Batf2 is expressed in immune cells such as T cells, B cells,^3^ macrophages, and dendritic cells.^4,5^ Batf2 was originally identified as an inhibitor of AP-1 via its interaction with c-JUN in cancer cells.^6^ We previously reported that Batf2 associates with Irf1 to induce inflammatory responses in IFN-γ and LPS-activated macrophages.^4^ Knockdown of *Batf2* by shRNA in IFN-γ or LPS-activated macrophages caused significant reduction of important early immune response genes (*Tnf*, *Ccl5*, and *IL12b*), including the bacterial effector, killing gene *Nos2*. More recently, another group further elucidated a role for IFN-γ- induced Batf2 in mediating IL-17 production and tissue damage during *T. cruzi* infection.^7^ Additionally, in absence of Batf3, Batf2 compensates for the development of CD103^+^ DCs in mice,^5^ a subset of DCs reported, suppressing helminth-driven immunity through constitutive expression of IL-12.^8^

In this study, we provide evidence for the regulation of immunity to Type 1 and Type 2 infectious diseases by Batf2. Using human whole blood transcriptomics, we identified elevated expression of *BATF2* as an early correlate for tuberculosis (TB) disease progression in adolescents with latent *Mycobacterium tuberculosis* (Mtb) infection. We further explored the role of Batf2 in a loss of function approach using Mtb-infected *Batf2*-deficient mice in comparison to infected wild-type mice. Interestingly, *Batf2*^*−/*−^ mice were highly resistant to TB disease exhibiting reduced tissue inflammation, pulmonary histopathology, and subsequently increased survival during acute infection. Mechanistically, we identified Batf2 as a transcriptional inducer of inflammatory responses during Mtb infection in mice and showed that BATF2 is a predictive biomarker for TB disease in humans in a prospective cohort study in adolescents. Similarly, Batf2 deficiency ameliorated the outcome of murine *Listeria monocytogenes* (Lm) infection by reducing bacterial burden and associated tissue inflammation. In contrast, Batf2 was important to limit untoward immune responses and small intestinal fibro-granulomatous inflammation during murine schistosomiasis. Together, our data reveal a regulatory role of Batf2 on the host immune responses to Type 1 (TB and listeriosis) and Type 2 (schistosomiasis) diseases.

## Results

### *Batf2*^−*/*−^ mice are resistant to the hypervirulent HN878 strain of Mtb with reduced acute lung inflammation

**Fig. 1:**
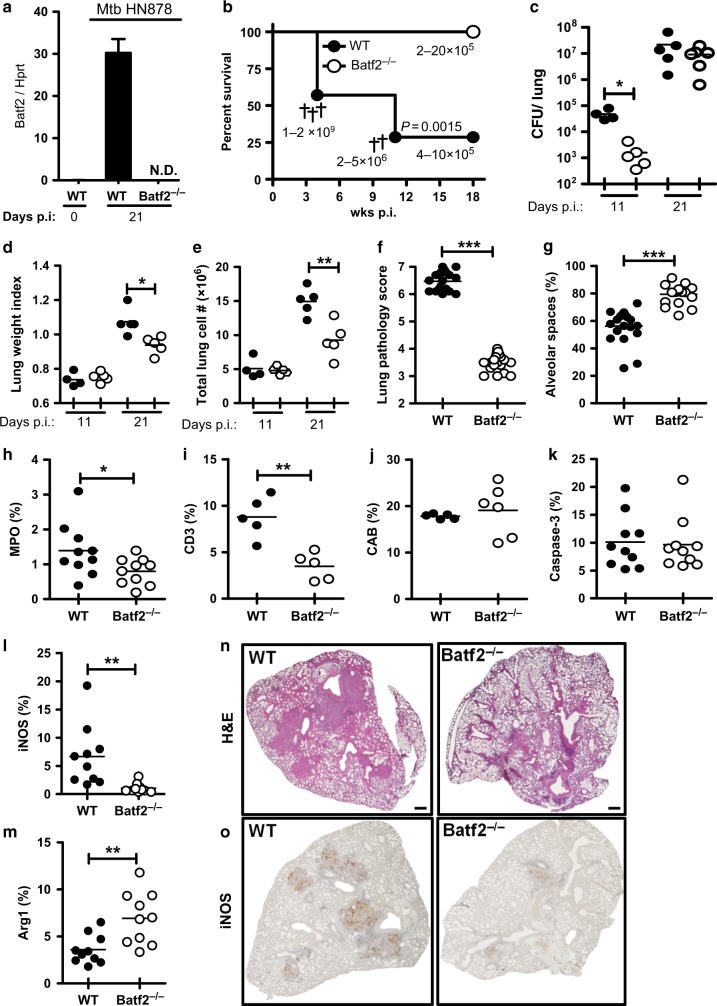
Batf2 deletion renders mice resistant to hypervirulent Mtb HN878 infection with concomitant reduced pulmonary inflammation. Control littermates (WT) and *Batf2*^*−/*−^ mice were infected intranasally with 100 CFU/mouse of Mtb HN878 (*n* = 5 mice/group). **a** Mice were killed at 3 weeks post-infection to sort CD11c^+^Siglec-F^+^autofluorescence^high^ alveolar macrophages by flow cytometry for *Batf2* mRNA expression relative to Hprt housekeeping gene by RT-PCR. **b** For survival study, mice were infected with 350 CFU/mouse of Mtb HN878 (*n* = 7–10 mice/group). Lung bacterial burden of Mtb HN878-infected mice are shown with indicated CFU/lung from moribund mice and mice that were killed at the termination of the experiment. Kaplan–Meier survival analysis with log-rank test *P* = 0.0015, WT vs. *Batf2*^*−/*−^. Mice were killed at 11 days and 3 weeks post-infection (inoculation dose: 100 CFU/mouse) to determine **c** lung CFU burden, **d** lung weight index, and **e** lung cell numbers. **f** At 3 weeks post infection, lung histopathology scores were graded from 1–10 in 4 deep cut H&E lung sections per mice (30 µm apart) and based on perivascular/peribronchiolar lymphocytic infiltrates, reduced ventilated alveolar spaces and extensive pulmonary lesions. **g** Alveolar spaces at 3 weeks post-infection, were quantified from 4 deep cut H&E lung sections per mice (30 µm apart). **h–m** The percentage of positive MPO, CD3, and CAB (Chromotrope Aniline Blue), Caspase-3, iNOS, and Arg1 staining per lung section was quantified from 1–2 deep cut lung sections per mice at 3 weeks p.i. (30 µm apart). **n**, **o** Representative histopathology sections (×10 magnification) at 3 weeks post infection for H&E and iNOS (scale bar = 400 µm). Error bars denote mean ± SEM. Data shown are representative of 2–4 independent experiments. *P < 0.05; **P < 0.01; ***P < 0.001; N.D not detected, Student’s *t*-test

In a genome-wide transcriptomics analysis, we previously reported that Batf2 is highly induced in Mtb-infected and IFN-γ activated macrophages (M1) in vitro.^4^ Lung alveolar macrophages are the first host cells that become infected by Mtb.^9^ Thus, we first determined *Batf2* mRNA expression in flow-sorted alveolar macrophages (CD11c^+^Siglec-F^+^autofluorescence^high^) from HN878 Mtb-infected WT and *Batf2*^*−/*−^ mice at 3 weeks post-infection. *Batf2* mRNA expression was detected in wild-type alveolar macrophages (Fig. 1a). As expected Mtb-infected *Batf2*^*−/*−^ mice did not express *Batf2* mRNA in alveolar macrophages.

To explore the consequence of a Batf2 deficiency in Mtb infection *in vivo*, a lethal dose of hypervirulent Mtb HN878 (350 CFU/mouse) was intranasally administered to *Batf2*^*−/*−^ mice and control littermates (WT). Subsequent mortality was observed in control littermates, whereas no deaths were observed in *Batf2*^*−/*−^ mice (Fig. 1b). Strikingly, 71.4% of the control littermates died during the experiment, whereas all infected *Batf2*^*−/*−^ mice survived up to 18 weeks post infection. Kaplan-Meier analysis demonstrated significant survival differences (*P* = 0.0015) between WT and *Batf2*^*−/*−^. Of interest, control mice started to die as early as 4 weeks post-infection with a high pulmonary bacterial burden from 1–2 × 10^9^ CFU, whereas *Batf2*^*−/*−^ mice at the termination of the experiment had only 2–20 × 10^5^ CFU (3–4 log reduced) pulmonary bacterial burden (Fig. 1b).

To further analyze the role of Batf2 in TB, mice were infected with a sub-lethal Mtb HN878 intranasal dose (100 CFU/mouse). CFU counts were significantly reduced in *Batf2*^*−/*−^ when compared to WT mice at 11 days but similar at 3 weeks post-infection (Fig. 1c). At 3 weeks post infection, *Batf2*^*−/*−^ mice resulted in significantly reduced lung weight index, total lung cell numbers, lung pathology score, as well as histopathology (H&E) in the lungs compared to WT mice (Fig. 1d-f, n). Lung inflammation was also quantified by measuring the free alveolar spaces, which demonstrated increased ventilated spaces in *Batf2*^*−/*−^ mice, indicative of reduced pulmonary lesions in *Batf2*^*−/*−^, compared to WT mice (Fig. 1g). Neutrophil influx into lungs was also reduced in *Batf2*^*−/*−^ mice, quantified by tissue-damaging factor myeloperoxidase (MPO) staining (Fig. 1h). Also, T cell recruitment measured by CD3 staining was significantly reduced in *Batf2*^*−/*−^ lung sections when compared to WT mice (Fig. 1i). In contrast, CAB (Chromotrope Aniline Blue) staining indicating fibrotic tissues were similar between both groups (Fig. 1j). Furthermore, reduced cell numbers and decreased inflammation observed in *Batf2*^*−/*−^ lungs was not due to increased apoptosis, but rather attributed to decreased cellular recruitment, hence Caspase-3 staining, a marker for apoptosis^10^ was similar between both groups (Fig. 1k). INOS, the enzyme producing the anti-mycobacterial effector molecule nitric oxide, was significantly reduced with a concomitant increase in Arg1, a marker for alternatively activated macrophages in *Batf2*^*−/*−^ lungs (Fig. 1l, m, o). Together, these results suggest that the presence of Batf2 during sub-lethal Mtb infection has no influence on bacterial burdens in the lungs but strikingly increases detrimental histopathology due to increased pulmonary inflammation and lesion size.

### Batf2 induces pro-inflammatory responses in lung recruited macrophages, leading to deleterious inflammation and TB disease progression

**Fig. 2:**
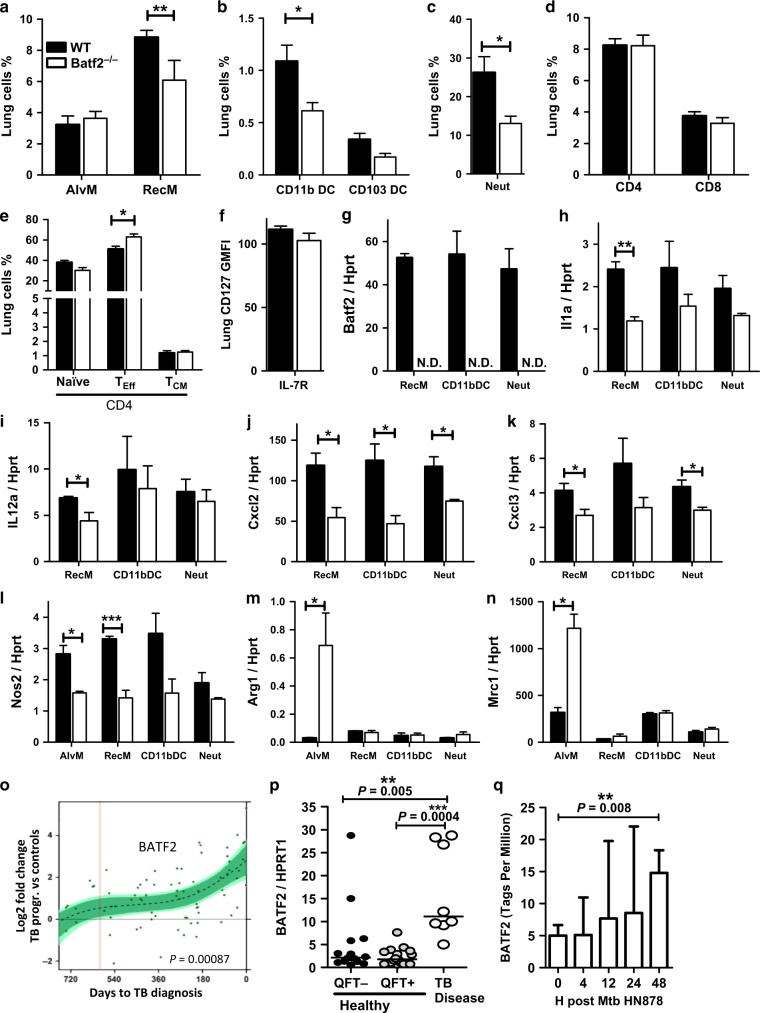
Reduced pro-inflammatory macrophage responses in *Batf2*^*−/*−^ lungs following Mtb HN878 infection and increased *BATF2* expression in human whole blood as a correlate for TB risk progression. Control littermates (WT) and *Batf2*^*−/*−^ mice were infected intranasally with 100 CFU/mouse of Mtb HN878 (*n* = 5 mice/group) and mice were killed at 3 weeks post-infection. Percentages of lung **a** CD11c^+^SiglecF^+^autofluorescence^high^ alveolar macrophages and CD11b^+^ F4/80^+^Ly6G^-^ interstitial recruited macrophages; **b** CD11b^+^Cd11c^+^MHCII^+^CD103^-^Ly6C^−^ and CD11b^−^CD103^+^CD11c^+^ DC; **c** CD11b^+^Ly6G^+^ neutrophils; **d** CD3^+^CD4^+^, CD3^+^CD8^+^ T cells; **e** CD44^low^CD62L^high^ naïve CD4^+^, central memory CD44^high^CD62L^high^ CD4^+^, effector CD44^high^CD62L^low^ CD4^+^ T cells, and **f** GMFI of CD127^+^ in CD3^+^CD4^+^ T cells. **g–n** CD11b^+^ F4/80^+^Ly6G^−^ interstitial recruited macrophages, CD11b^+^CD11c^+^MHCII^+^CD103^−^ DC, CD11b^+^Ly6G^+^ neutrophils and CD11c^+^SiglecF^+^autofluorescence^high^ alveolar macrophages were sorted by flow cytometry to determine mRNA expression of *Batf2, IL1a*, *IL12a*, *Cxcl2*, *Cxcl3*, *Nos2*, Arg1, and Mrc1. **o** BATF2 expression was determined by RNA sequencing in TB progressors in comparison to healthy non-progressor participants and plotted as log2 fold changes (FC) versus days before TB disease. The dotted line represents spline fit to the overall dataset, and the green shading represents the statistical analysis of the data, done by fitting a nonlinear spline with three degrees of freedom to the fold change values. **p** *Batf2* mRNA expression relative to HPRT1 housekeeping gene was determined by RT-PCR from whole blood of healthy, QuantiFERON Gold TB In-Tube negative (QFT^-^) and positive (QFT^+^) adolescents, and participants diagnosed with active tuberculosis (TB) disease (One-way ANOVA with Tukey’s multiple comparison tests). **q** Human monocyte-derived macrophages were infected with Mtb HN878 to measure BATF2 expression by CAGE transcriptomics. Error bars denote mean ± SEM for **a**–**n** and median with interquartile range for **p** and **q**. Data shown in **a**–**n** are representative of two independent experiments. *P < 0.05; **P < 0.01; ***P < 0.001; Student’s *t*-test

To better define cellular infiltration in the lungs, cell populations were analyzed by flow cytometry, at 3 weeks post-infection with 100 CFU/mouse of Mtb HN878. Number and percentages of CD11b^+^F4/80^+^Ly6G^−^ interstitial recruited macrophages, CD11b^+^CD11c^+^MHCII^+^CD103^−^ Ly6C^−^ DC and CD11b^+^Ly6G^+^ neutrophils in the lungs were significantly lower in *Batf2*^*−/*−^ mice compared to WT (Fig. 2a–c and Fig. S1A-C). T lymphocyte population in the lungs and the mediastinal lymph nodes were marginally affected by the absence of *Batf2* (Fig. 2d–f and Fig. S1D and E). FACS sorting of interstitial recruited macrophages, CD11b^+^ DC’s and neutrophils (Fig. S1K, gating strategy) from Mtb-infected wild-type mice showed similar mRNA levels of *Batf2* during infection (Fig. 2g). Of importance, recruited interstitial macrophages from Mtb-infected *Batf2*-deficient mice resulted in reduced transcription of several pro-inflammatory cytokines, as well as chemokines, such as *Il1a*, *Il12a*, *Cxcl2*, and *Cxcl3*, compared to WT control cells (Fig. 2h–k). In addition, *Nos2* was significantly reduced in *Batf2*^*−/*−^ compared to WT alveolar and recruited macrophages (Fig. 2l). In contrast, Arg1 and Mrc1, makers for alternatively activated macrophages were significantly increased from sorted alveolar macrophages in *Batf2*^*−/*−^ compared to WT controls (Fig. 2m, n), confirming the switch from iNOS to Arginase expression in the lung *Batf2*^*−/*−^ mice. Non-infected naïve WT control and *Batf2*-deficient mice displayed similar baseline lung immune cell responses (Fig. S2), with non-detectable levels of MPO, iNOS, and Arg1. These results suggest that Batf2 is involved in early inflammatory responses, with recruiting interstitial macrophages following Mtb infection, increasing early TB disease.

We recently identified a mRNA expression signature of the risk of TB in whole blood that differentiated infected adolescents who progressed to TB disease from adolescents who remained healthy.^11^ Among the 16 signature genes, *BATF2* was significantly increased over time (during progression from latent Mtb infection to TB disease (Fig. 2o). The light green shading represents 99% CI of the spline fit and where this deviates from 0, the data show statistically significant upregulation of *BATF2*. This analysis confirmed a highly significant upregulation in *BATF2* expression over time (*p*-value: 0.00087) at a false discovery rate of 0.035. Moreover, participants with active TB disease demonstrated striking increased *BATF2* mRNA (Fig. 2p) when compared to healthy controls (both QFT^−^and QFT^+^ *P* = 0.0004) by RT-PCR. Additionally, Mtb HN878 infection in human monocyte-derived macrophages from healthy donors also showed significant increased *BATF2* expression at 48 h post-infection (Fig. 2q). Comparing to other infections or inflammatory conditions, human whole blood *BATF2* expression was significantly increased in patients with Influenza A, B, rhinovirus infection (Public US Cohort GSE 68310) and sarcoidosis (Public UK Cohort GSE 42826) when compared to healthy controls (Fig. S1J). Collectively, this suggests that elevated Batf2 expression is an excellent indicator of host inflammation that also depicts TB disease progression.

### *Batf2*^*−/*−^ mice are resistant to primary and secondary listeriosis

**Fig. 3:**
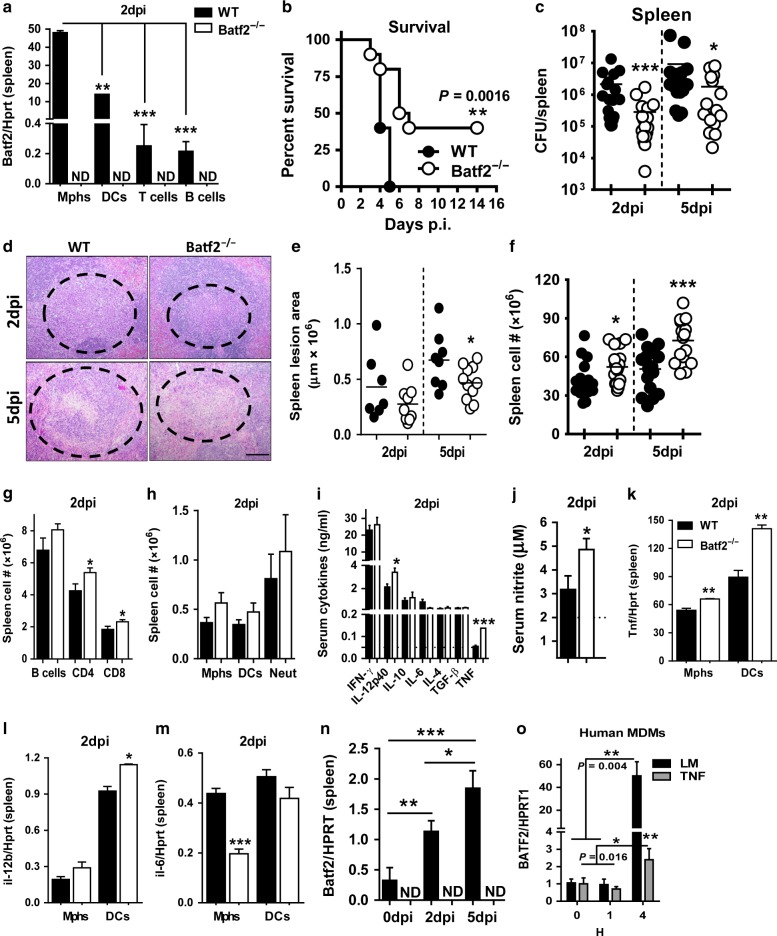
Increased survival, reduced burdens and tissue pathology in *Batf2*^*−/*−^ mice following *Listeria monocytogenes* infection and increased Batf2 expression in human macrophages. **a** Mice were infected with *Listeria monocytogenes* (Lm) 2 × 10^5^ CFU/mouse. At 2 days post infection, *Batf2* mRNA expression was determined in flow-sorted macrophages (CD11c^−^CD11b^+^MHCII^+^), dendritic cells (CD11b^−^CD11c^+^MHCII^+^), T cells (CD3^+^CD4^+^) and B cells (CD3^−^CD19^+^) from spleen of WT and *Batf2*^*−/*−^ mice. **b** WT and *Batf2*^*−/*−^ mice were infected intraperitoneally with *Listeria monocytogenes* (Lm) 2 × 10^5^ LM CFU/mouse (*n* = 9–10 mice/group). Kaplan–Meier survival analysis with log-rank test *P* = 0.0016, WT vs. *Batf2*^*−/*−^ respectively. **c** Mice were killed at day 2 and 5 post-infection to determine bacterial loads in the spleens. **d** Representative histopathological images and **e** lesion quantifications in the spleen were analyzed with 3 deep cuts of H&E sections per mice (30 µm apart; scale bar = 100 µm). **f** A total number of splenocytes from WT and *Batf2*^*−/*−^ mice at the indicated time points after Lm infection. **g** Numbers of CD3^+^CD4^+^, CD3^+^CD8^+^ T cells and **h** macrophages (CD11c^−^CD11b^+^MHCII^+^), dendritic cells (CD11b^−^CD11c^+^MHCII^+^) and neutrophils (CD11c^−^CD11b^+^Gr-1^+^) cells at day 2 after infection. **i** IFN-γ, IL-12p40, TNF, IL-6, IL-4, IL-10, and TGF-β and **j** nitrite production was measured in serum of infected mice at 2 days after Lm infection. The dotted line represents the limit of the detection of ELISA. **k** *Tnf*, **l** *Il-12b*, and **m** *IL-6* mRNA transcripts in flow-sorted splenic macrophages and dendritic cells from day 2 Lm-infected mice. **n** *Batf2* mRNA expression was measured in the total spleen cells of WT naïve mice (0 days), 2 days and 5 days after Lm infection. **o** Human monocyte-derived macrophages were infected with Lm or stimulated with TNF (10 ng/ml) to measure *Batf2* mRNA expression relative to HPRT1 housekeeping gene by quantitative RT-PCR. Error bars denote mean or mean ± SEM. Data shown are representative of two experiments or pooled from four independent experiments. *P < 0.05; **P < 0.01; ***P < 0.001; N.D not detected, Student’s *t*-test, unpaired

We further explored whether Batf2 may have a wider importance in Type 1 infectious diseases using experimental murine listeriosis. We uncovered that *L. monocytogenes* infection induces *Batf2* mRNA expression particularly abundant in macrophages followed by the dendritic cells in the spleen (Fig. 3a) and liver (Fig. S3A). In addition, Batf2 mRNA expression was also detected in T cells and B cells to a lesser extent in spleen (Fig. 3a), though absent in liver (Fig. S3A). In naïve mice, *Batf2* mRNA expression was also higher in macrophages (Fig. S4A), however greatly reduced when compared to Lm-infected mice (Fig. 3a), suggesting *Batf2* expression was indeed driven by Lm infection predominantly in macrophages and dendritic cells. Consistent with TB, Lm-infected *Batf2*^*−/*−^ mice were also more resistant during high- (Fig. 3b) and low-dose (Fig. S3B) infection with increased survival and reduced bacterial burden in the spleen (Fig. 3c) and liver (Fig. S3C), compared to WT mice. Moreover, Lm-infected *Batf2*^*−/*−^ mice presented decreased histopathological inflammation, with reduced lesions in spleen (Fig. 3d, e) and liver (Fig. S3D, E) when compared to WT mice, a result consistent with TB lung inflammation. Total spleen cell counts were significantly increased in Lm-infected *Batf2*^*−/*−^ mice compared to control mice (Fig. 3f), whereas liver cells were significantly reduced at day 5 after Lm infection (Fig. S3F). At 2-day post-infection, T cell numbers (CD4^+^ and CD8^+^) in spleen (Fig. 3g) and B cells in addition to CD4 + T cells in the liver (Fig. S3G were increased in *Batf2*^*−/*−^ mice, whereas myeloid cell populations such as macrophages, dendritic cells, and neutrophils remain unaffected in the spleen (Fig. 3h). Similarly, macrophages and dendritic cells had showed no differences, however, interestingly neutrophils were significantly increased in the liver (Fig. S3H). Importantly, neither the total cell numbers (Fig. S4B) nor immune cell populations were affected in spleen (Fig. S4C, D) and liver (Fig. S4E, F) of naïve mice. Furthermore, IL-12p40 and TNF (Fig. 3i), both important for Lm resistance^12,13^ were significantly increased in the sera of Lm-infected *Batf2*^*−/*−^ mice, as well as nitric oxide, an important effector molecule to clear Lm infection (Fig. 3j).^14^ However, at tissue level reduced IFN-γ, IL-12p40, and TNF (Fig. S3I) in liver presented reduced hepatic inflammation. Furthermore, other pro-inflammatory and tissue protective cytokines such as IL-10, IL-4, and TGF-β were similar between the groups during Lm infection (Fig. 3i and Fig. S3I). These cytokines were also unaffected in the absence of Batf2 in both sera (Fig. S4G) and liver (Fig. S4H) tissues as well as, serum nitric oxide (Fig. S4I) in naïve mice, suggesting cytokine differences were not due to gene deficiency at homeostasis. At the cellular level, *Batf2*-deficient macrophages and dendritic cells-derived *Tnf* was increased in spleen (Fig. 3K) and dendritic cells in the liver (Fig. S3J). In addition to *Tnf*, transcripts of *Il12b* were significantly increased in splenic dendritic cells (Fig. 3l) whereas *Il6* transcripts were decreased in macrophages (Fig. 3m). In liver macrophages and dendritic cells, both *Il12b* (Fig. S3K) and *Il6* (Fig. S3L) transcripts remained unaffected between WT and *Batf2*^*−/*−^ mice. As expected, both *Il12b* and *Il6* transcripts were not detected in macrophages and dendritic cells sorted from spleen and liver tissues of naïve mice. *Tnf* transcripts in macrophages and dendritic cells were however similar between WT and *Batf2*^*−/*−^ mice (Fig. S4J), suggesting a deficiency of Batf2 had no major effect at mRNA level under homeostasis.

We then asked whether Lm infection drives *Batf2* mRNA expression in mice. Indeed, *Batf2* expression was increased in spleen (Fig. 3n) and liver (Fig. S3M) during listeriosis. Since *BATF2* mRNA expression was increased during active TB disease (Fig. 2o, p) and in Mtb-infected human macrophages (Fig. 2q), we then asked whether Lm infection would also increase *BATF2* expression in human macrophages. Indeed, *BATF2* mRNA expression was significantly increased in human macrophages at 4 h after Lm infection (Fig. 3o). To directly test whether this increase was specific to Lm, we stimulated macrophages with TNF. At 4 h post stimulation, *BATF2* mRNA expression was increased (Fig. 3o), however it was ~25-fold lower when compared to Lm infection, suggesting Lm drives the induction of *Batf2* mRNA expression at a greater extent in human macrophages. Furthermore, we asked whether the absence of Batf2 plays a role in memory to Lm infection, which would rapidly clear infection during the secondary challenge. Increased T cell responses, owing to their memory of Lm antigens during the primary challenge, are responsible for enhanced protection during secondary Lm challenge.^15^ In *Batf2*^*−/*−^ mice, a secondary Lm infection was more rapidly controlled with reduced bacterial burdens in the liver and spleen at 2 and 4 days after infection (Fig. S3N), suggesting that the absence of Batf2 might also enhance the ability of T cells to directly and/or indirectly control secondary Lm infection. Altogether, our findings showed that Batf2 plays a deleterious key role in the host immunity against listeriosis.

### Batf2 deficiency aggravates small intestinal fibro-granulomatous inflammation in *S. mansoni*-infected mice and increases the small intestinal permeability

**Fig. 4:**
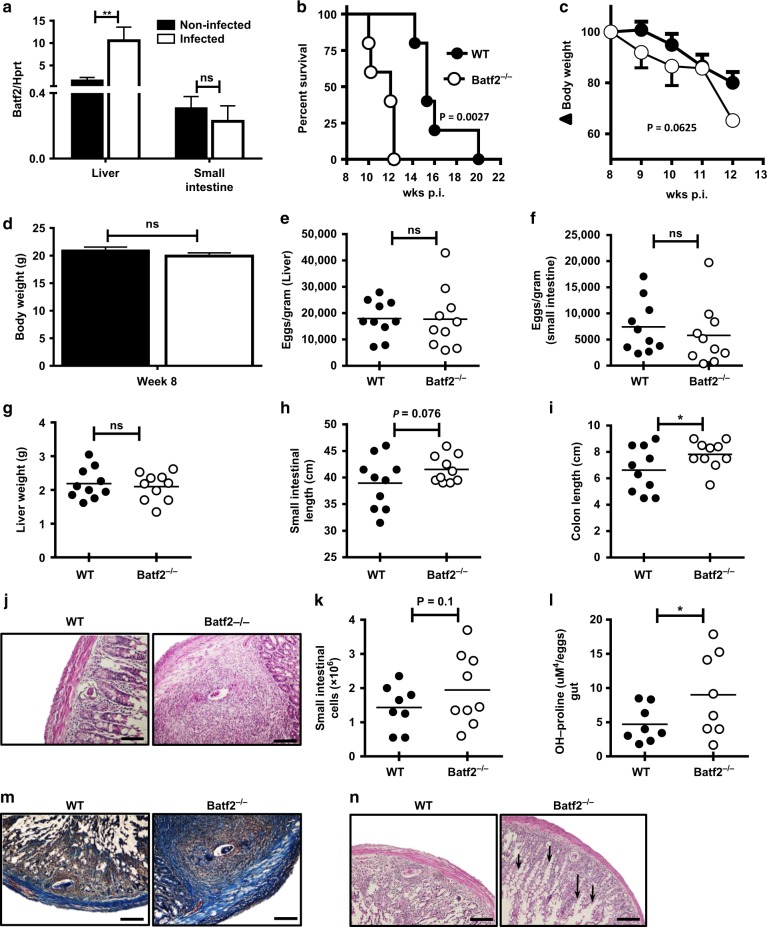
Batf2 deficiency drives an increased fibro-granulomatous inflammation in the small intestine of *S. mansoni* infected mice. Control littermates (WT) and *Batf2*^*−/*−^ mice were percutaneously infected with 80 live *S. mansoni* cercariae and were killed at 8 weeks post-infection. Liver and small intestinal tissues were collected from naïve and 8 weeks infected WT and *Batf2*^*−/*−^ mice. **a** *Batf2* mRNA expression relative to Hprt housekeeping gene by RT-PCR to quantify *Batf2* mRNA levels. **b** Representative survival rate and **c** body weight change of mice post-infection measured each week up to week 12. **d** Summary of body weight at week 8 post-infection. Mice were killed at 8 weeks post-infection to determine the number of *S. mansoni* eggs lodged in the liver (**e**) and small intestinal **f** tissues, **g** the liver weight in grams, the length of the **h** small intestine (from base of stomach to beginning of cecum) and **i** colon respectively in cm. **j** Representative H&E staining of small intestinal sections (scale bar = 200 µm). **k** Number of small intestinal cells from animals 8 weeks post-infection. **l** Levels of hydroxyproline and **m** representative CAB staining of small intestinal sections (scale bar = 200 µm) as a measure of fibrosis. **n** Representative H&E staining for analysis of small intestinal tissue integrity (scale bar = 200 µm). Error bars denote mean ± SEM. Data shown are representative of one to three independent experiments with a sample size of *n* = 8–10 mice per group. *p < 0.05, **p < 0.01, and ***p < 0.001 vs WT using one-tailed Student’s *t*-test, with survival measured using Log-rank (Mantel-Cox) test, and the body weight change measured using Wilcoxon Signed-Rank test. ns not significant

The targeting of Batf2 to alter the outcome of infectious diseases is attractive but still poorly informed strategy.^1^ Having convincingly demonstrated a deleterious role for Batf2 during Type-1-dominated by infectious diseases, we next sought to question the role of this transcription factor during Type-2 infectious diseases. To do so, we used a murine model of acute schistosomiasis (bilharzia). *Batf2*^*−/*−^ mice and WT controls were infected percutaneously with high dose (80–100) of *Schistosoma mansoni* cercariae and immune responses were analyzed at 8 weeks post-infection in the liver and small intestine, where the highly immunogenic and pathogenic *S. mansoni* eggs preferentially lodge. We first noted that *Batf2* mRNA expression was significantly increased in the liver of *S. mansoni*-infected animals, while expression remained unchanged in the small intestine of the infected mice (Fig. 4a), when compared to the mRNA expression in the naïve control tissues. In fact, Batf2 deficiency rendered animals more susceptible to acute schistosomiasis (Fig. 4b) associated with a rapid wasting disease (Fig. 4c) that was not confounded by a differential body weight in animals from both groups (Fig. 4d). Intriguingly, however, egg burden in liver (Fig. 4e) and small intestinal tissues (Fig. 4f) remained similar between WT and *Batf2*^*−/*−^ mice. As either liver and/or small intestinal pathology usually drive host susceptibility to schistosomiasis morbidity,^16,17^ we further investigated for indications of liver or small intestinal pathology in these mice, as we reasoned that differential pathology in the absence of differential parasite burden might explain the susceptibility of *Batf2*^*−/*−^ mice during acute schistosomiasis. Batf2 deficiency did not prompt any major aggravation of the liver pathology as determined by the measure of hepatomegaly (Fig. 4g) during acute schistosomiasis. However, we noted a non-significant increase in the small intestine (Fig. 4h) and a significant increase in colon length (Fig. 4i) in the absence of Batf2, indicating a possible crucial role of this transcription factor in the small intestinal pathophysiology during *S. mansoni* infection. Cellular infiltration around the trapped eggs defined by microscopic observation (Fig. 4j), as well as by determination of total tissue cell counts (Fig. 4k) was increased in the small intestine of *Batf2*-deficient animals, indicating a heightened small intestinal inflammation in *Batf2*-deficient mice during acute schistosomiasis. Moreover, the absence of Batf2 led to a strikingly more advanced egg-surrounding small intestinal fibrosis in *Batf2*^*−/*−^ mice compared to wild-type controls as demonstrated by both hydroxyproline levels (Fig. 4l) and CAB staining (Fig. 4m). Notably, histological assessment of macrophage activation markers revealed an unaffected arginase expression profile (Fig. S5A, B) amid a drastic reduction of iNOS expression (Fig. S5C, D). This suggests a reduced ability of small intestinal macrophages, undergoing classical macrophage M1 activation during schistosomiasis in the absence of Batf2. Consistent with our previously defined key role of Batf2 in the promotion of M1 macrophage activation and function.^4^ Our present observation supports a necessary role for Batf2 in the host ability to mount an M1 activation profile in the small intestine during acute schistosomiasis. Liver analyses did not show such a pathological profile in *Batf2*^*−/*−^ mice as we observed no altered granulomatous response and a diminished fibrotic response around trapped eggs in the liver of Batf2^*−/*−^ mice during acute schistosomiasis (Fig. S5E-H). This suggests the small intestine was the primary site of pathology in our model. Notably, despite baseline differences in cellularity and responses (Fig. S6), such a fibro-pathological profile was not apparent in the small intestine of naïve Batf2^*−/*−^ animals (Fig. S7A-F). This suggest a defect in response to infection rather than just an intrinsic defect as a driver of the observed profile in *S. mansoni*-infected *Batf2*^*−/*−^ mice. In support, small intestinal tissue TNF-alpha levels as well as serum TNF-alpha levels were elevated in *S. mansoni* infected *Batf2*^*−/*−^ mice (Fig. S7G, H). Furthermore, histological assessment of small intestinal sections from schistosomiasis-diseased animals showed several opened areas stretching from the lumen to the epithelium leading to loss of intestinal villi in the small intestine of Batf2^*−/*−^ mice (Fig. 4n).^18^ Collectively, these data suggest that the absence of Batf2 results in an exacerbated small intestinal fibro-granulomatous inflammation associated with a wasting disease thus premature death during the Type-2 dominated model of acute schistosomiasis.

### Batf2 deficiency results in heightened cellular and cytokine responses in the small intestine during acute schistosomiasis

**Fig. 5:**
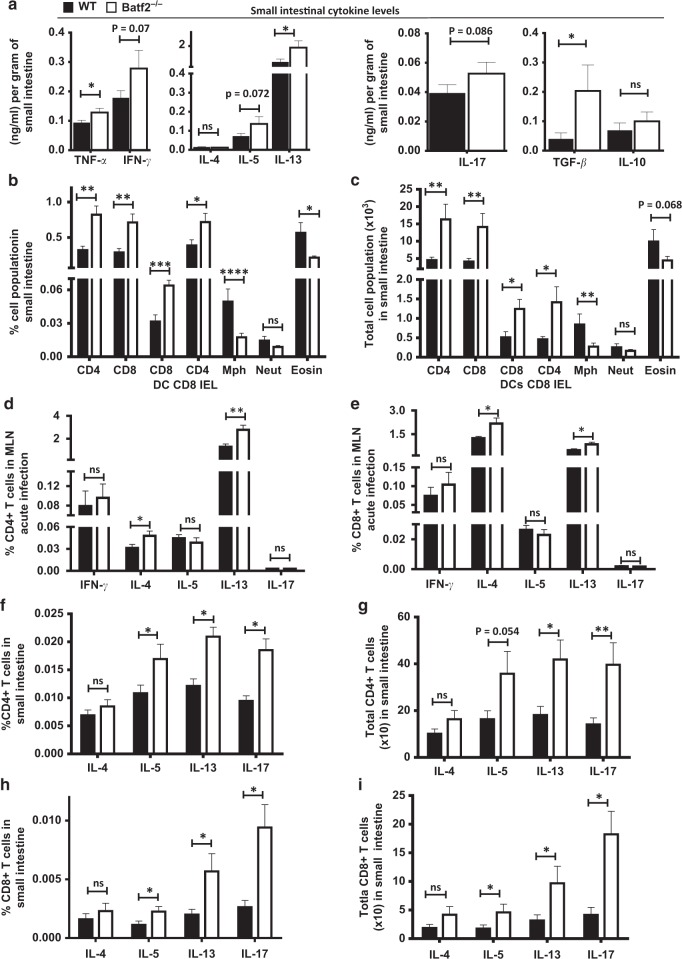
Batf2 deficiency drives heightened cellular inflammatory responses in the small intestine of *S. mansoni* mice. Control littermates (WT) and *Batf2*^*−/*−^ mice were percutaneously infected with 80 live *S. mansoni* cercariae and were killed at 8 weeks post infection. **a** Concentrations of small intestinal cytokine levels normalized to mg of tissue were determined using ELISA. Flow cytometry was used to determine percentages (**b**) and absolute numbers (**c**) of CD4^+^ intra-epithelial lymphocytes (IEL), CD8^+^ IEL, CD4^+^ CD8^+^ IEL, CD8^+^ dendritic cells, neutrophils (CD11b^+^ Ly6G^+^), macrophages (CD11b^+^ F4/80^+^), and eosinophils (CD11b^+^ Siglec-F^+^), **d** percentage of IFN-γ CD4^+^, IL-4^+^ CD4^+^, IL-5^+^ CD4^+^, IL-13^+^ CD4^+^, IL-17^+^ CD4^+^ T cells in MLN. **e** Percentage of IFN-γ CD4^+^, IL-4^+^ CD8^+^, IL-5^+^ CD8^+^, IL-13^+^ CD8^+^, IL-17^+^ CD8^+^ T cells in MLN. **f** Percentage and **g** cell numbers of IL-4^+^ CD4^+^, IL-5^+^ CD4^+^, IL-13^+^ CD4^+^, IL-17^+^ CD4^+^ IEL in small intestine. **h** Percentage and **i** cell numbers of IL-4^+^ CD8^+^, IL-5^+^ CD8^+^, IL-13^+^ CD8^+^, IL-17^+^ CD8^+^ IEL in small intestine. Error bars denote mean ± SEM. Data shown are representative of one to three independent experiments with a sample size of *n* = 8–10 mice per group. *p < 0.05, **p < 0.01, and ***p < 0.001 vs WT using one tailed Student’s *t*-test. ns not significant

We next defined the immune profile that associates with a Batf2 deficiency in the small intestine of *S. mansoni*-infected mice. In the absence of Batf2, there was a significant elevation in the amounts of TNF-α, IL-13, TGF-β and a moderate elevation of IFN-γ, IL-5, and IL-17 (Fig. 5a) in the small intestinal tissue of infected mice. Analyses on the immune cell dynamics in small intestinal tissues showed an expansion of CD4^+^, CD8^+^, and CD4^+^ CD8^+^ intraepithelial lymphocytes (IELs), and CD8^+^ DCs in percentage (Fig. 5b) and total cell numbers (Fig. 5c). Intracellular cytokine detection in these expanded cells showed an elevated production of pro-fibrotic cytokines, i.e. IL-5, IL-13, and IL-17 by MLN and small intestinal tissue CD4^+^ and CD8^+^ T cells (Fig. 5d–i), including the regulatory cytokine IL-10 by MLN CD4^+^ and CD8^+^ T cells (Fig. S7I, J), from Batf2-deficient mice when compared to the same cells from littermate controls (Fig. 5d–i). This aligned with elevated cytokine production observed in the small intestinal tissue of *S. mansoni*-infected Batf2-deficient mice (Fig. 5a). Furthermore, the transcription factors associated with polarization of CD4 T cells (Fig. S7K, L) indicated a general increase of inflammatory response (both Th1 and Th2-related as per the analyzed indicators) rather than a preferential increase of a T helper arm of the immune response in the absence of Batf2. Altogether, these findings demonstrate that Batf2 is required by the host to control T helper mediated inflammatory responses in the small intestine during acute schistosomiasis.

## Discussion

Here, we show that absence of Batf2 could dampen over-inflamed immunological state leading to resistance against tuberculosis and listeriosis in mice. Deletion of Batf2 in mice displayed enhanced survival rate when compared to control mice that succumbed to hyper-virulent Mtb (HN878) infection. Survival correlated with decreased pulmonary inflammation, revealed by reduced and compact granulomas, which contained similar bacillary loads amongst the groups. We also showed that *Batf2* expression in lung macrophages progressively increased following a virulent HN878 strain of Mtb infection, a clinical isolate known to induce inflammation.^19,20^ This showed that Batf2 exerts its inflammatory signature through lung macrophages since flow-sorted macrophages from Mtb-infected *Batf2*^*−/*−^ mice had significantly reduced pro-inflammatory chemokine (*Cxcl2, Cxcl3*), cytokine responses (*Il1a*, *IL12*), and killing effector molecule (*Nos2*), when compared to macrophages from wild-type mice. Interestingly, our findings in *Batf2*^*−/*−^ mice mirrored mice lacking the receptor for type 1 IFN,^21^ which also resulted in reduced early lung inflammation with smaller lesions. In addition, despite higher bacterial burden (10^5^ CFUs), IFNar1^*−/−*^ mice were resistant to Mtb infection by decreasing recruitment of inflammatory macrophages, chemokines, pro-inflammatory cytokines (*Il1a*, *IL1b*, *Tnf*, *Il6*) and decreased nitric oxide killing effector function. It seems that Mtb aggravates inflammation to develop a persistence replication niche in the host. As TB disease progresses, the accumulation of inflammatory cells drives lung tissue pathology and allows for a permissive state where Mtb can replicate. Indeed, granulocyte depletion significantly extended the survival of Mtb-infected mice^22^ and TB susceptibility is determined by the increased accumulation of a permissive monocyte/macrophage population in the lung.^23^ The mechanisms of increased resistance of *Batf2*^*−/*−^ mice could be explained by reduced recruitment of inflammatory macrophages in the lungs, thereby limiting the numbers and availability of permissive macrophages.

Previously, it was reported that *Batf3*-deficient mice displayed similar survival rates to Erdman strain of Mtb infection compared to wild-type animals.^5^ In contrast to Batf3, Batf2 deletion in mice controlled lung pathology and ill-defined granuloma formation that leads to an aggressive TB pathology/damage, which drives the disease progression and subsequent death of the host following HN878 infection. Therefore, the inflammatory response observed during Mtb infection needs to be balanced and an over-inflamed state of immune activation may lead to TB disease and tissue pathology. This balance between pro- and anti-inflammatory signals can be observed spatially within lung granulomas during Mtb infection.^24^

In addition to TB, infection with *Listeria monocytogenes* (Lm) also resulted in increased Batf2 expression, as a function of time in spleen but transient in liver tissue. Furthermore, flow-sorted splenic cell populations revealed that *Batf2* mRNA expression was dominated by macrophages followed by dendritic cells, T cells and B cells during Lm infection in mice. The absence of Batf2 also resulted in an increased production of systemic host protective cytokines (TNF, IL-12) in sera and enhanced recruitment of T and B lymphocytes in mice. This was evident in the observed increased survival rate associated with reduced bacilli loads and decreased spleen and liver histopathology in *Batf2*^*−/*−^ mice. Similarly, Lm infection in mice deficient for Batf3 showed enhanced survival due to the depletion of CD8α and CD103^+^ DCs, obligate entry point populations, which are required for the progressive infection.^25^ In contrast to Lm infection, Batf3^−/−^ mice were highly susceptible to *Salmonella enterica serovar Typhimurium* (ST) infection to their reduced ability to produce inflammatory cytokines (TNF, IL-6, and IL-1a) and chemokines (MIP-1a, MIP-1b) by CD8α DCs, which is required for local CD8 + T cell priming to clear early infection.^26^ Notably, both Lm and ST are fast proliferating and cytopathic intracellular pathogens, yet the progression of these diseases was different in *Batf3*^−/−^ mice (amelioration in LM versus aggravation in ST). Considering this, the observed discrepancies in the inflammatory responses between Lm and Mtb infection models are therefore unsurprising, given the differential proliferating profile of both pathogens in the course of an infection, as Lm is rapidly proliferating but Mtb slow growing. Nevertheless, we also found similarities in both infectious models in Batf2^*−/*−^ mice where inflammatory responses appeared decreased at the tissue levels (liver in Lm and lung in Mtb). Of note, the immune cell responses such as pro-, anti-inflammatory, and Th2 cytokines (lung/serum/liver/intestine/gut), as well as numbers and percentages of immune cell populations (lung/spleen/liver/intestine) remained unaffected between naïve WT and Batf2^*−/*−^ mice. This suggested that these responses were not biased due to gene deletion at homeostasis (Fig. S2, S4, S6). Apart from murine studies, BATF2 expression was decreased during the clinical stage of human lung cancer.^27^ In contrast to human lung cancer, using South African Adolescent Cohort Study,^11^ we identified increased *BATF2* expression in whole blood of asymptomatic individuals who progressed to active TB disease. Moreover, Batf2 expression was also increased upon Mtb and Lm infection in macrophages derived from healthy human donors. Hence, this showed that Batf2 expression is detrimental in murine Mtb and Lm infections. This matches our conclusions from mouse studies and implies that BATF2 can be used as a predictive marker of disease progression in Type 1 diseases.

Intriguingly, however, Batf2 deficiency aggravated small intestinal fibrogranulomatous responses during acute murine schistosomiasis. This indicates a host requirement of Batf2 to control small intestinal fibrosis in our Th2-dominated setting. Given that excessive small intestinal fibrosis is a widely reported pathology that mediates the burden of several diseases, such as intestinal schistosomiasis,^28–30^ inflammatory bowel disease^31^ or ulcerative colitis,^32^ our finding of a protective role of Batf2 in this context is of potentially wider value. Our data showed a counter-regulatory role of this factor in the advancement of small intestinal fibrosis during schistosomiasis. We also observed higher levels of TNF-alpha together with disrupted intestinal wall which both point towards a possible role of Batf2 in inducing increased small intestinal permeability during acute schistosomiasis^33–36^ but this is still to be addressed experimentally. In fact, our subsequent observations of elevated cytokine release by small intestinal cells together with an elevated recruitment of intestinal cells in *Batf2*-deficient mice during acute schistosomiasis, further point towards an anti-inflammatory/pathological role of this factor in the small intestine of mice during acute schistosomiasis. Of note, the expansion of small intestinal CD8^+^ dendritic cells primarily associated with the deleterious effect of Batf2 deficiency during acute schistosomiasis, which does suggests a role for small intestinal CD8^+^ dendritic cells – and most likely the resulting of elevated CD4^+^ and CD8^+^ T cell responses – in the increased small intestinal fibrogranulomatous inflammation reported. The expansion of CD8^+^ DCs in *Batf2*^*−/*−^ mice indicates a regulatory role for Batf2 on small intestinal CD8^+^ DCs during inflammation.^5^ Such a regulatory role of Batf2 on inflammatory DCs is consistent with the recently reported inhibitory role of Batf2 on Th17-inducing DCs during *T. cruzi* infections.^7^ It remains clear, therefore, that Batf2 deficiency unleashes a pro-inflammatory and cytokine-rich small intestine environment around the trapped *S. mansoni* eggs, therefore, emphasizing on an anti-inflammatory role of this factor in the small intestine of *S. mansoni* infected animals. Altogether, our findings therefore strongly argue against a therapeutic target for the BATF2 blockade in the context of Type-2 controlled schistosomiasis in agreement with its recently reported anti-inflammatory role against pathological tissue Th17 responses during *T. cruzi* infection.^7^

It was shown that Batf2 is expressed in dendritic, monocyte, natural killer, and T cells.^37^ In our study, we report on a differential inflammatory T cell response in *Batf2* knockout mice when compared to WT mice suggesting that some of the observations in these cancer models are possibly due to a role of Batf2 in T cells. Consistent with the previously reported higher expression rate of *Batf2* by CD8^+^ T cells when compared to CD4^+^ T cells, our present data reveal a more pronounced inflammatory surge in CD8^+^ cells within the T cells compartment of *Batf2*-deficient mice during acute schistosomiasis. Further studies on the intrinsic dysregulation of CD8^+^ T cells in the absence of Batf2 independently from antigen-presenting myeloid cells might be needed, for more clarity on the subject. As of now, our present data might support a stronger regulatory need of this factor in CD8^+^ T cells, compared to CD4^+^ T cells. This might explain the opposing role of the factor in Type-1 and Type-2 diseases, given the differential influence of CD8^+^ T cells in such diseases.^38^

Overall, whilst a pathogenic role is clear for BATF2 in the context of Type-1 infectious diseases and warrants further assessment of the therapeutic value of inhibiting this factor, Type-2 and Type-17 controlled infectious diseases appears to require BATF2 to tame untoward immune responsiveness and as such might be aggravated by the Batf2 blockade. In the light of our present findings, Batf2 is unprecedentedly presented as a fine and versatile regulator of tissue infectious immunepathologies and caution is therefore recommended in strategies aiming at targeting Batf2 to ameliorate Type-2 infectious diseases.

## Materials and methods

### Mice

Batf2-deficient mice (*Batf2*^*−/*−^) were generated in 129S6/SvEvTac-derived EDJ22 embryonic stem cells^5^ and heterozygous mice (Batf2^+/-^) were purchased by Jackson Laboratories (USA), subsequently intercrossed to generate Batf2^*−/*−^ and littermate control 129S6/SvEv mice at the Animal Research Facility, University of Cape Town.

### Ethics statement

All animal experiments were performed in accordance with the South African National Standard (SANS 10386:2008) and University of Cape Town of practice for laboratory animal procedures. The protocol for Mtb (012/036), Lm (015/037), and Sm (016/027) were approved by the Animal Ethics Committee, University of Cape Town. The human Adolescent Cohort Study^11,39^ was approved by the Human Research Ethics Committee (045/2005), University of Cape Town.

### Mtb, Lm, and *S. mansoni* infection and determination of burdens in mice

Anaesthetized mice were infected intranasally with 25 μl of viable HN878 Mtb bacilli into each nasal cavity with doses of 100 CFU/mouse for immune response analysis and 350 CFU/mouse for mortality studies. Bacterial loads, histopathological and flow cytometry analyses in lungs of Mtb-infected mice were determined as previously described.^40^ The lung weight index calculation was performed as a measure of inflammatory infiltration using: square root [(Lung weight in mg/Mouse weight in g)*10]/10. Mice were infected intraperitoneally with *Listeria monocytogenes* (Lm) with a high-dose of 2 × 10^5^ CFU/mouse and a low-dose of 3 × 10^4^ CFU/mouse. Tissue burdens, cytokine analysis, and histopathology performed as previously described.^41^ Briefly, aseptically harvested lungs (Mtb) and liver/spleen (Lm) were homogenized in 0.01% Tween-PBS and 10-fold dilutions were plated on 7H10 (Mtb) and tryptic soy (Lm) agar plates for the determinations of CFUs. Mice were infected percutaneously with 80 live *S. mansoni* cercariae parasites obtained from infected *Biomphalaria glabrata* snails (a gift from Adrian Mountfold, York, UK). Eggs were purified from digested sections of liver or ileum of infected animals as previously described.^42^

### Quantitative real-time RT-PCR

Total murine RNA was reverse transcribed by Transcriptor First Strand cDNA Synthesis Kit (Roche) according to manufacturer’s instructions. Real-time PCR was performed with LightCycler® 480 SYBR Green I Master mix in LightCycler® 480 II (Roche). From the human adolescent cohort study,^43^ RNA was extracted from PAXgene tubes and cDNA synthesis was performed with SuperScript II Reverse Transcriptase (Life Technologies).

### Histology

Lungs (for Mtb), liver and spleen (for Lm), and liver and small intestinal (for Sm) sections were collected from euthanized mice and placed in 4% formaldehyde solutions. Embedded sections were then stained with hematoxylin and eosin (H&E) or chromotrope aniline blue (CAB). The percentage of free alveolar spaces was defined as the open spaces in whole lung sections in relation to the total lung tissue area. Both free spaces and total tissue areas were measured using the area measurement tool by the Nikon microscope imaging software NIS-elements and the % of alveolar space was calculated in Excel. A blinded quantification was performed to measure the percentage of MPO, CAB, CD3, iNOS Arg1, and Caspase 3 using the Nikon microscope imaging software NIS-elements. The diameters of each granuloma containing a single egg were measured using a computerized morphometry analysis program as previously described.^17^

### Hydroxyproline assay

A modified protocol was employed to measure hydroxyproline levels in tissue as previously described.^17,44^ Briefly, weighed tissue samples were hydrolyzed and the supernatant was neutralized with 1% phenolphthalein and titrated against 10M NaOH. An aliquot was mixed with isopropanol and added to a chloramine-T/citrate buffer solution (pH 6.0) (Sigma). Ehrlich’s reagent solution was added and measured at 570 nm. Hydroxyproline levels were calculated by using 4-hydroxy-l-proline (Calbiochem) as standard, and results were expressed as μg hydroxyproline per weight of tissue that contained 10^4^ eggs.

### Tissue homogenate and ELISA

Intestinal tissue samples were supplemented with extraction buffer (1× PBS buffer with 0.2 g of a protease inhibitor, and 0.1% Tween), and subjected to homogenization for 30 s with 15 s intervals. The samples were then centrifuged at 5000 rpm for 5 minutes at 4 °C, and the supernatant was collected in new Eppendorf tubes. The protein content was measured using BCA assay according to the manufacturer’s instructions (Thermo Scientific). Standard ELISA was then performed on the homogenized samples. From the concentrations obtained by ELISA, each cytokine concentration obtained was normalized to the amount of protein initially measured in a sample. Normalized cytokine concentration = initial cytokine concentration (ng/ml)/total protein content measured per sample in gram.

### Isolation of small intestinal cells by enzymatic digestion adapted and optimized studies

The small intestine was cut out from the abdomen and single cells were isolated as previously described with minor modifications.^45–47^ Briefly, the contents of the excised small intestine were flushed out and the tissue washed in 1X PBS buffer. The tissue was then chopped into fine pieces and then re-suspended into 5 ml digestion buffer solution (220 U/mg Collagenase I and 13 U/mg DNAse I in 50 ml DMEM medium supplemented with 5% iFCS). The samples were then incubated at 37 °C on a shaker for 30 min. Following incubation, the samples were passed through a 100 µm and 70 µm sieves, then centrifuged at 1200 rpm, for 10 min in 4 °C and re-suspended in 3 ml 1xPBS + 3% FCS. The samples were then supplemented with 1.7 ml isotonic Percoll (9 vol Percoll + 1 vol 10× PBS) and mixed thoroughly by inverting gently. The samples were then centrifuged for 500*g*, for 10 min at 4 °C without brakes. The supernatant was carefully removed and the pellets were re-suspended in 5 ml medium (IMDM + 10% iFCS and 0.5% Pen-strep) and sieved through a 40 µm sieve. The samples were centrifuged at 1200 rpm, 10 min at 4 °C and then re-suspended in 1 ml of the medium. The cells were assessed for viability and counted using an electron microscope.

### Flow cytometry

The following antibodies were used for flow cytometry analysis: CD4, CD8, CD11b, CD11c, Siglec-F, CD45, IL-4, IL-5, IL-17 purchased from BD Bioscience; F4/80, IL-13 purchased from Affymetrix eBioscience; Ly6G purchased from Sony Biotechnology Inc; 7AAD purchased from Sigma. Cells were re-stimulated non-specifically with a mitogenic cocktail of PMA-Ionomycin and monensin (to block cytokine secretion) for 6–8 h. The cells were stained for the surface, intracellular and intranuclear markers identifying cells, secreted cytokines, and associated transcription factors. The stained cells were then acquired on an LSR Fortessa machine (BD Immunocytometry system) and data were analyzed using Flowjo software (Treestar).

### Whole blood RNA-Seq from the Adolescent Cohort Study

RNA-Seq was performed on whole blood samples from 46 progressors and 107 non-progressor controls as previously described.^11,39^ *BATF2* expression was determined in this cohort by computing a 99% confidence intervals (CI) for the temporal trends by performing 2000 iterations of spline fitting after bootstrap resampling from the full dataset.

### Statistical analysis

All data were analyzed using GraphPad Prism v 6.0, a Student's *t*-test (two-tailed with unequal variance) or unless otherwise stated in Fig. legends. Means are shown as ± SEM., *P < 0.05, **P < 0.01 and ***P < 0.001 respectively.

## Electronic supplementary material

Supplementary Figures legends

Supplementary Figures
