# Supplementary material for: Batf2 differentially regulates tissue immunopathology in Type 1 and Type 2 diseases
Source: Mucosal Immunol. 2018 Dec 12;12(2):390–402. doi: 10.1038/s41385-018-0108-2 (PMC7051910; doi:10.1038/s41385-018-0108-2)
Supplement: Supplementary file 1 — Supplementary Figures legends [file 41385_2018_108_MOESM1_ESM.docx]

**Supplementary Figure Legends:**

**Fig. S1. Lung cell numbers and unaltered lymphocyte cell populations in mediastinal lymph nodes following Mtb HN878 infection in *Batf2^-/-^* deficient mice.** Control littermates (WT) and *Batf2^-/-^* mice were infected intranasally with 100 CFU/mouse of Mtb HN878 (n=5 mice/group) and mice were sacrificed at 3 weeks post-infection. (**A**) Cell numbers of lung CD11c^+^SiglecF^+^autofluorescence^high^ alveolar macrophages and lung CD11b^+^ F4/80^+^Ly6G^-^ interstitial recruited macrophages; (**B**) lung CD11b^+^CD11c^+^MHCII^+^CD103^-^ Ly6C^-^ and lung CD11b^-^CD103^+^CD11c^+^ DC; (**C**) lung CD11b^+^Ly6G^+^ neutrophils; Percentages (**D and F**) and numbers (**E and G**) of mediastinal lymph node CD3^+^CD4^+^, CD3^+^CD8^+^ T cells, CD44^low^CD62L^high^ naïve CD4+, central memory CD44^high^CD62L^high^ CD4^+^, effector CD44^high^CD62L^low^ CD4^+^ T cells and (**H**) GMFI of MLN CD127^+^ in CD3^+^CD4^+^ T cells. (**I**) Mtb-infected mice were sacrificed at 24 hours post infection to determine the CFU lung uptake. (**J**) Human BATF2 expression from publically available datasets from whole blood of healthy controls (HC) and patients with Influenza A, B, rhinovirus infection (US Cohort GSE 68310) and sarcoidosis (UK Cohort GSE 42826). (**K**) Gating strategy of lung sorted cell populations. Error bars denote mean ± SEM. Data shown are representative of two independent experiments. *, P < 0.05; **, P < 0.01. Student’s t-test, unpaired.

**Fig. S2. Similar naïve baseline lung immune cell responses between non-infected WT control and Batf2-deficient mice.** Naïve non-infected control littermates (WT) and *Batf2^-/-^* mice (n=8 mice/group) were sacrificed to determine (**A**) lung weight index and (**B**) lung cell numbers. (**C**) Alveolar spaces were quantified from 3 deep cut H&E lung sections per mice (30 µm apart). (**D**) The percentage of positive Caspase-3, per lung section, was quantified. (**E-K**) Percentages and cell numbers of lung CD11c^+^SiglecF^+^autofluorescence^high^ alveolar macrophages, lung CD11b^+^ F4/80^+^Ly6G^-^ interstitial recruited macrophages, lung CD11b^+^CD11c^+^MHCII^+^CD103^-^ Ly6C^-^, lung CD11b^-^CD103^+^CD11c^+^DC, lung CD11b^+^Ly6G^+^ neutrophils and lung GMFI of CD127. (**L-O**) Percentages and numbers of lung CD3^+^CD4^+^, CD3^+^CD8^+^ T cells, CD44^low^CD62L^high^ naïve CD4+, central memory CD44^high^CD62L^high^ CD4^+^, effector CD44^high^CD62L^low^ CD4^+^ T cells. (**P and Q**) CD11b^+^ F4/80^+^Ly6G^-^ interstitial recruited macrophages, CD11b^+^CD11c^+^MHCII^+^CD103^-^ DC and CD11b^+^Ly6G^+^ neutrophils were sorted by flow cytometry to determine mRNA expression of *Batf2* and *Nos2.* Error bars denote mean ± SEM. *, P < 0.05; **, P < 0.01. Student’s t-test, unpaired.

**Fig. S3. Batf2 deletion resulted in decreased liver burdens, inflammation in primary infection and increased bacterial clearance during secondary Lm infection.** (**A**) Mice were infected with *Listeria monocytogenes* (Lm) 2x10^5^ CFU/mouse. At 2 days post infection, *Batf2* mRNA expression was determined in flow-sorted macrophages (CD11c^-^CD11b^+^MHCII^+^) and dendritic cells (CD11b^-^CD11c^+^MHCII^+^) from the liver of WT and *Batf2^-/-^* mice. (**B**) WT and *Batf2^-/-^* mice were infected intraperitoneally with a low dose of 3x10^4^ LM CFU/mouse (n=9-10 mice/group). Kaplan-Meier survival analysis with log-rank test P = 0.017, WT vs. *Batf2^-/-^* respectively. (**C**) Mice were sacrificed at day 2 and 5 post-infection to determine bacterial loads in the livers. (**D**) Representative histopathological images and (**E**) lesion quantification in the liver was analyzed with 3 deep cuts of H&E sections per mice (30 µm apart). (**F**) A total number of liver cells harvested from WT and *Batf2^-/-^* mice at the indicated time points after Lm infection. (**G**) Numbers of CD3^+^CD4^+^, CD3^+^CD8^+^ T cells and B cells (CD3^-^CD19^+^) and (**H**) macrophages (CD11c^-^CD11b^+^MHCII^+^), dendritic cells (CD11b^-^CD11c^+^MHCII^+^) and neutrophils (Cd11c^-^ CD11c^+^Gr-1^+^) cells at day 2 after infection. (**I**) Liver homogenates were analyzed for IFN-γ, IL-12p40, TNF IL-6, IL-4, IL-10 and TGF-β production by ELISA. (**J**) *Tnf*, (**K**) *Il-12b* and (**L**) *Il-6* mRNA transcripts in flow-sorted splenic macrophages and dendritic cells from day 2 Lm-infected mice. (**M**) *Batf2* mRNA expression was measured in the total liver cells of WT naïve mice (0 days), 2 days and 5 days after Lm infection. (**N**) Bacilli burdens of Listeria in spleen and liver in mice sacrificed on day 2 and day 4 after secondary challenge with LM. Error bars denote mean ± SEM. Data shown are representative of two independent experiments. *, P < 0.05; **, P < 0.01; ***, P < 0.001; Student’s t-test.

**Fig. S4. Deletion of Batf2 has no effect under homeostatic conditions in mice.** (**A**) *Batf2* mRNA expression was determined in flow-sorted naïve macrophages (CD11c^-^CD11b^+^MHCII^+^), dendritic cells (CD11b^-^CD11c^+^MHCII^+^), T cells (CD3^+^CD4^+^) and B cells (CD3^-^CD19^+^) from spleen of WT and *Batf2^-/-^* mice. (**B**) A total number of liver and spleen cells harvested from WT and *Batf2^-/-^* naïve mice. (**C-F**) Numbers of CD3^+^CD4^+^, CD3^+^CD8^+^ T cells, B cells (CD3^-^CD19^+^), macrophages (CD11c^-^CD11b^+^MHCII^+^), dendritic cells (CD11b^-^CD11c^+^MHCII^+^) and neutrophils (Cd11c^-^ CD11b^+^Gr-1^+^) cells in (**C-D**) spleen and (**E-F**) liver of naïve mice. (**G-I**) Serum and liver homogenates were analyzed for IFN-γ, IL-12p40, TNF, IL-6, Il-4, Il-10 and TGF-β production by ELISA and (**H**) nitric oxide in serum by Griess reagent assay. (**J**) *Tnf* mRNA transcripts in flow-sorted splenic and liver macrophages and dendritic cells from naive mice. Data shown as mean ± SEM of n = 6 mice/group.

**Fig. S5.** **Batf2 deficiency impairs iNOS production by small intestinal cells during acute schistosomiasis.** Control littermates (WT) and Batf2^-/-^ mice were percutaneously infected with 80 live *S. mansoni* cercariae and were sacrificed 8 weeks post infection. Representative arginase measurement using (**A**) immunohistochemistry and (**B**) automated computerised microscopy. Representative iNOS measurement using (**C**) immunohistochemistry (scale bar = 200 µm) and (**D**) automated computerised microscopy measurements. **(E**) Representative H&E staining (scale bar = 200 µm) analysis of granuloma development, and (**F**) summary of the granuloma sizes. (**G**) Representative CAB staining (scale bar = 200 µm), and (**H**) hydroxyproline levels as measures of fibrosis development. Error bars denote mean ± SEM. Data shown are representative of one to three independent experiments with a sample size of n = 8 - 10 mice per group. *p< 0.05, **p< 0.01, and ***p<0.001 vs WT using one tailed student’s t-test. ns, not significant.

**Fig. S6.** **Baseline levels of reported immunological features mediated during acute schistosomiasis.** Control littermates (WT) and Batf2-/- mice were sacrificed and (**A**) concentrations of small intestinal cytokine levels normalised to mg of tissue were determined using ELISA. Flow cytometry was used to determine percentages (**B**) and absolute numbers (**C**) of CD4^+^ intra-epithelial lymphocytes (IEL), CD8^+^ IEL, CD4^+^ CD8^+^ IEL, CD8^+^ dendritic cells, neutrophils (CD11b^+^ Ly6G^+^), macrophages (CD11b^+^ F4/80^+^), and eosinophils (CD11b^+^ Siglec-F^+^), (**D**) Percentage of IL-4^+^ CD4^+^, IL-5^+^ CD4^+^, IL-13^+^ CD4^+^, IL-17^+^ CD4^+^ T cells in MLN. (**E**) Percentage of IL-4^+^ CD8^+^, IL-5^+^ CD8^+^, IL-13^+^ CD8^+^, IL-17^+^ CD8^+^ T cells in MLN. (**F**) Percentage and (**G**) cell numbers of IL-4^+^ CD4^+^, IL-5^+^ CD4^+^, IL-13^+^ CD4^+^, IL-17^+^ CD4^+^ IEL in small intestine. (**H**) Percentage and (**I**) cell numbers of IL-4^+^ CD8^+^, IL-5^+^ CD8^+^, IL-13^+^ CD8^+^, IL-17^+^ CD8^+^ IEL in small intestine. Error bars denote mean ± SEM. Data shown are representative of one to three independent experiments with a sample size of n = 8 - 10 mice per group. *p< 0.05, **p< 0.01, and ***p<0.001 vs WT using one tailed student’s t-test. ns, not significant.

**Fig. S7.** **Baseline measures of the immunopathological features affected during schistosomiasis, and immune responses during acute schistosomiasis.** Liver weight in (**A**) grams and (**B**) percentage. The length of (**C**) small intestine (from the base of the stomach to the beginning of caecum), and (**D**) colon. (**E**) A number of small intestinal cells from animals and baseline level. (**F**) Levels of hydroxyproline in the small intestinal tissue as a measure of fibrosis. Concentrations of (**G**) small intestinal cytokine level normalized to mg of tissue and (**H**) serum cytokine levels determined using ELISA. (**I** and **J**) Percentage of IL-10^+^ CD4^+^, IL-10^+^ CD8^+^ in MLN during acute schistosomiasis. (**K** and **L**) Percentage of GATA3+ CD4+, and T-bet+ T cells in the small intestine. Error bars denote mean ± SEM. Data shown are representative of one to three independent experiments with a sample size of n = 8 - 10 mice per group. *p< 0.05, **p< 0.01, and ***p<0.001 vs WT using one tailed student’s t-test. ns, not significant.
