# Supplementary material for: Batf2 differentially regulates tissue immunopathology in Type 1 and Type 2 diseases
Source: Mucosal Immunol. 2018 Dec 12;12(2):390–402. doi: 10.1038/s41385-018-0108-2 (PMC7051910; doi:10.1038/s41385-018-0108-2)
Supplement: Supplementary file 2 — Supplementary Figures [file 41385_2018_108_MOESM2_ESM.pdf]

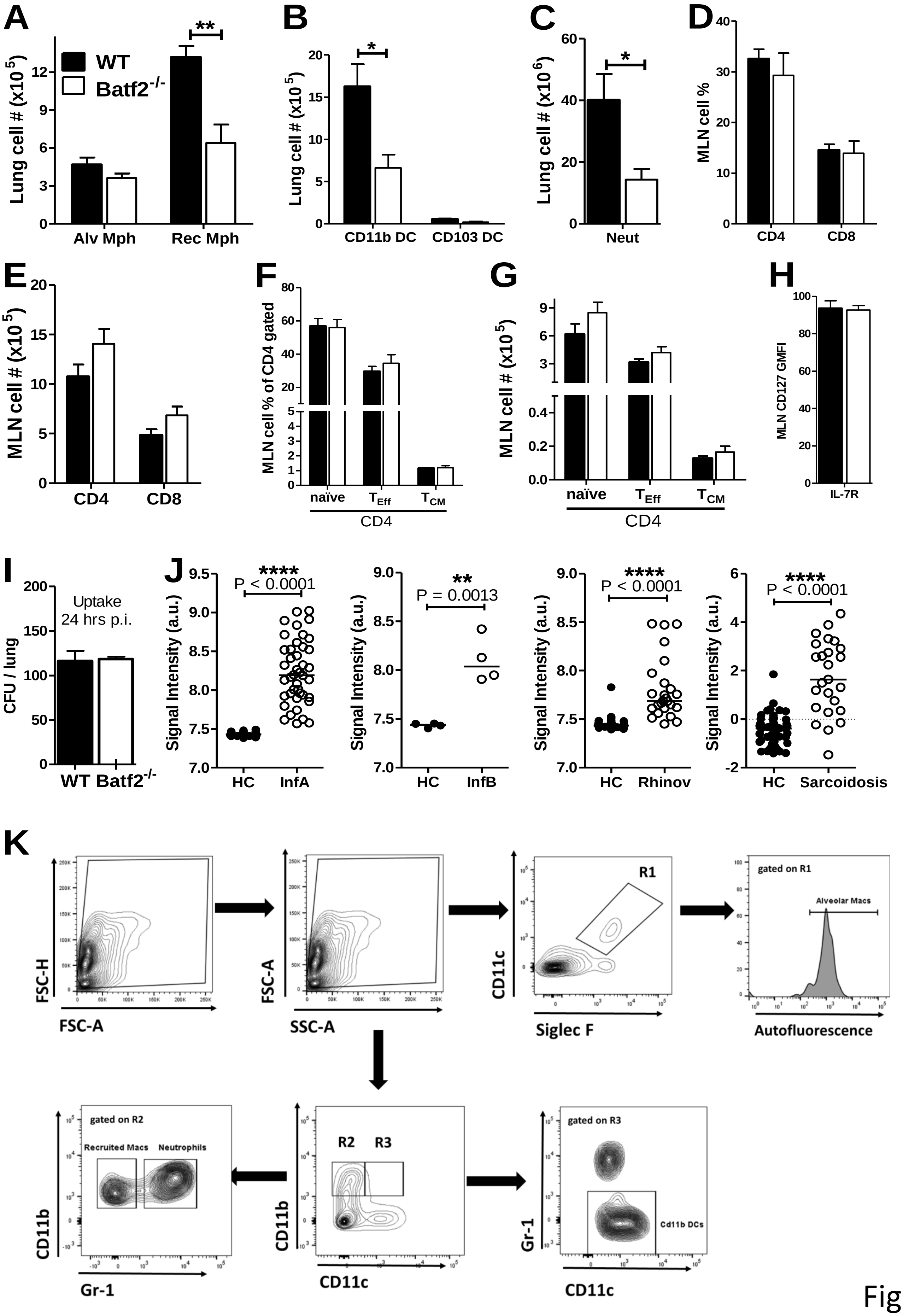
A
WT
Batf2-/-
Lung cell # (x10^5)
**
Alv Mph
Rec Mph
B
Lung cell # (x10^5)
*
CD11b DC
CD103 DC
C
Lung cell # (x10^6)
*
Neut
D
MLN cell %
CD4
CD8
E
MLN cell # (x10^5)
CD4
CD8
F
MLN cell % of CD4 gated
naïve
TEff
TCM
CD4
G
MLN cell # (x10^5)
naïve
TEff
TCM
CD4
H
MLN CD127 GMFI
IL-7R
I
Uptake
24 hrs p.i.
CFU / lung
WT
Batf2-/-
J
Signal Intensity (a.u.)
****
P < 0.0001
HC
InfA
**
P = 0.0013
HC
InfB
****
P < 0.0001
HC
Rhinov
****
P < 0.0001
HC
Sarcoidosis
K
FSC-H
FSC-A
SSC-A
CD11c
Siglec F
R1
gated on R1
Alveolar Macs
Autofluorescence
gated on R2
Recruited Macs
Neutrophils
CD11b
Gr-1
R2
R3
CD11c
gated on R3
Cd11b DCs

Fig. S1

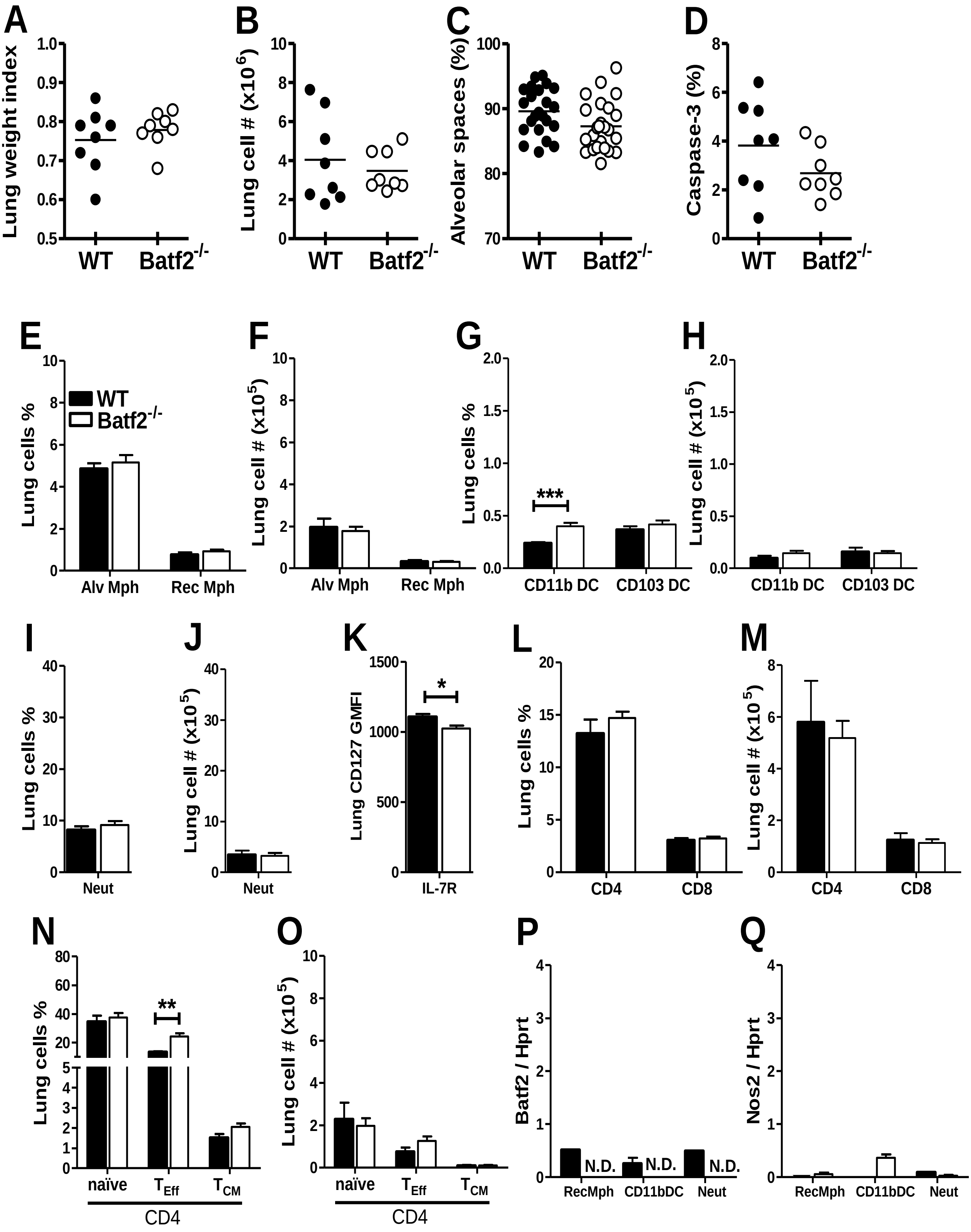

A
Lung weight index
WT
Batf2-/-
B
Lung cell # (x10^6)
C
Alveolar spaces (%)
D
Caspase-3 (%)
E
Lung cells %
Alv Mph
Rec Mph
F
Lung cell # (x10^5)
G
***
CD11b DC
CD103 DC
H
I
Neut
J
K
Lung CD127 GMFI
*
IL-7R
L
CD4
CD8
M
N
**
naïve
T_Eff
T_CM
O
P
Batf2 / Hprt
N.D.
RecMph
CD11bDC
Q
Nos2 / Hprt

Fig. S2

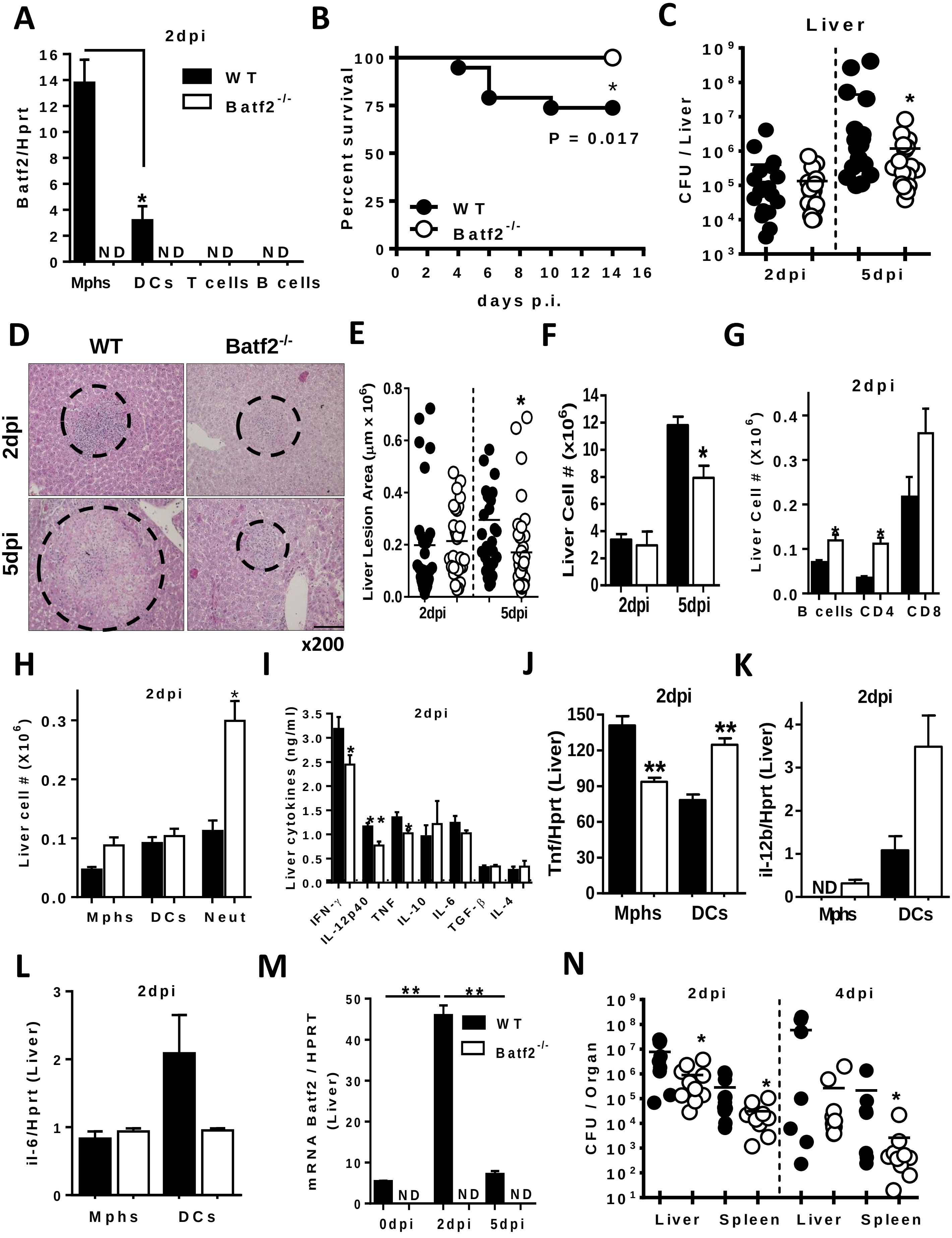
A
2dpi
Batf2/Hprt
WT
Batf2-/-
ND
ND
ND
ND
Mphs
DCs
T cells
B cells
B
Percent survival
P = 0.017
WT
Batf2-/-
days p.i.
C
Liver
CFU / Liver
2dpi
5dpi
D
WT
Batf2-/-
2dpi
5dpi
x200
E
Liver Lesion Area (μm x 10^6)
2dpi
5dpi
F
Liver Cell # (x10^6)
2dpi
5dpi
G
2dpi
Liver Cell # (X10^6)
B cells
CD4
CD8
H
2dpi
Liver cell # (X10^6)
Mphs
DCs
Neut
I
2dpi
Liver cytokines (ng/ml)
IFN-γ
IL-12p40
TNF
IL-10
IL-6
TGF-β
IL-4
J
2dpi
Tnf/Hprt (Liver)
Mphs
DCs
K
2dpi
il-12b/Hprt (Liver)
ND
Mphs
DCs
L
2dpi
il-6/Hprt (Liver)
Mphs
DCs
M
mRNA Batf2 / HPRT (Liver)
WT
Batf2-/-
ND
ND
ND
0dpi
2dpi
5dpi
N
2dpi
4dpi
CFU / Organ
Liver
Spleen
Liver
Spleen

Fig. S3

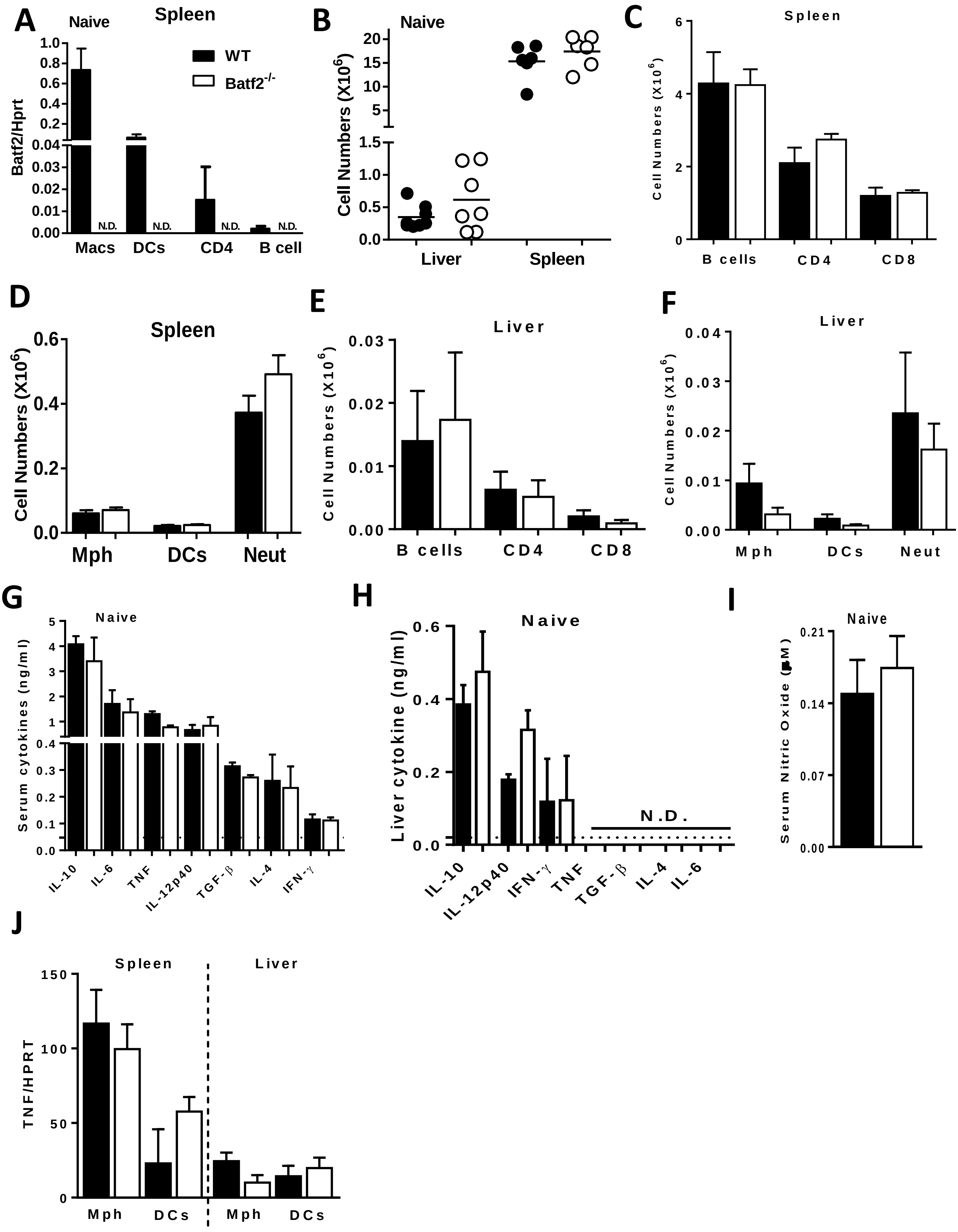
A
Naive
Spleen
WT
Batf2-/-
Batf2/Hprt
N.D.
Macs
DCs
CD4
B cell
B
Naive
Cell Numbers (X10^6)
Liver
Spleen
C
Spleen
Cell Numbers (X10^6)
B cells
CD4
CD8
D
Spleen
Cell Numbers (X10^6)
Mph
DCs
Neut
E
Liver
Cell Numbers (X10^6)
B cells
CD4
CD8
F
Liver
Cell Numbers (X10^6)
Mph
DCs
Neut
G
Naive
Serum cytokines (ng/ml)
IL-10
IL-6
TNF
IL-12p40
TGF-β
IL-4
IFN-γ
H
Naive
Liver cytokine (ng/ml)
N.D.
IL-10
IL-12p40
IFN-γ
TNF
TGF-β
IL-4
IL-6
I
Naive
Serum Nitric Oxide (μM)
J
Spleen
Liver
TNF/HPRT
Mph
DCs
Mph
DCs

Fig. S4

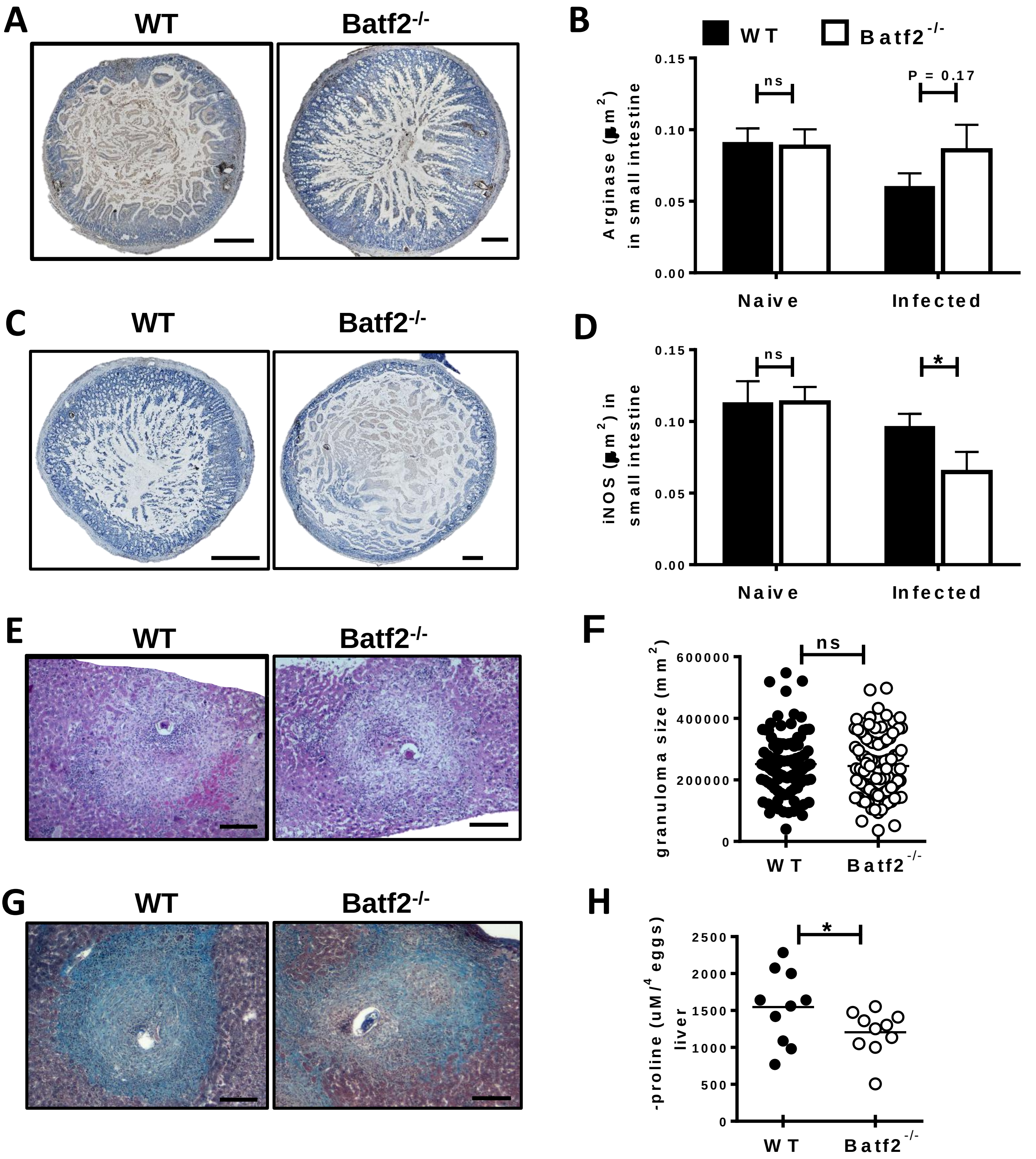
A
WT
Batf2-/-
B
WT
Batf2-/-
Arginase (µm²) in small intestine
0.15
0.10
0.05
0.00
ns
P = 0.17
Naive
Infected
C
WT
Batf2-/-
D
iNOS (µm²) in small intestine
0.15
0.10
0.05
0.00
ns
*
Naive
Infected
E
WT
Batf2-/-
F
granuloma size (mm²)
600000
400000
200000
0
ns
WT
Batf2-/-
G
WT
Batf2-/-
H
-proline (uM/⁴ eggs) liver
2500
2000
1500
1000
500
0
*
WT
Batf2-/-

Fig. S5

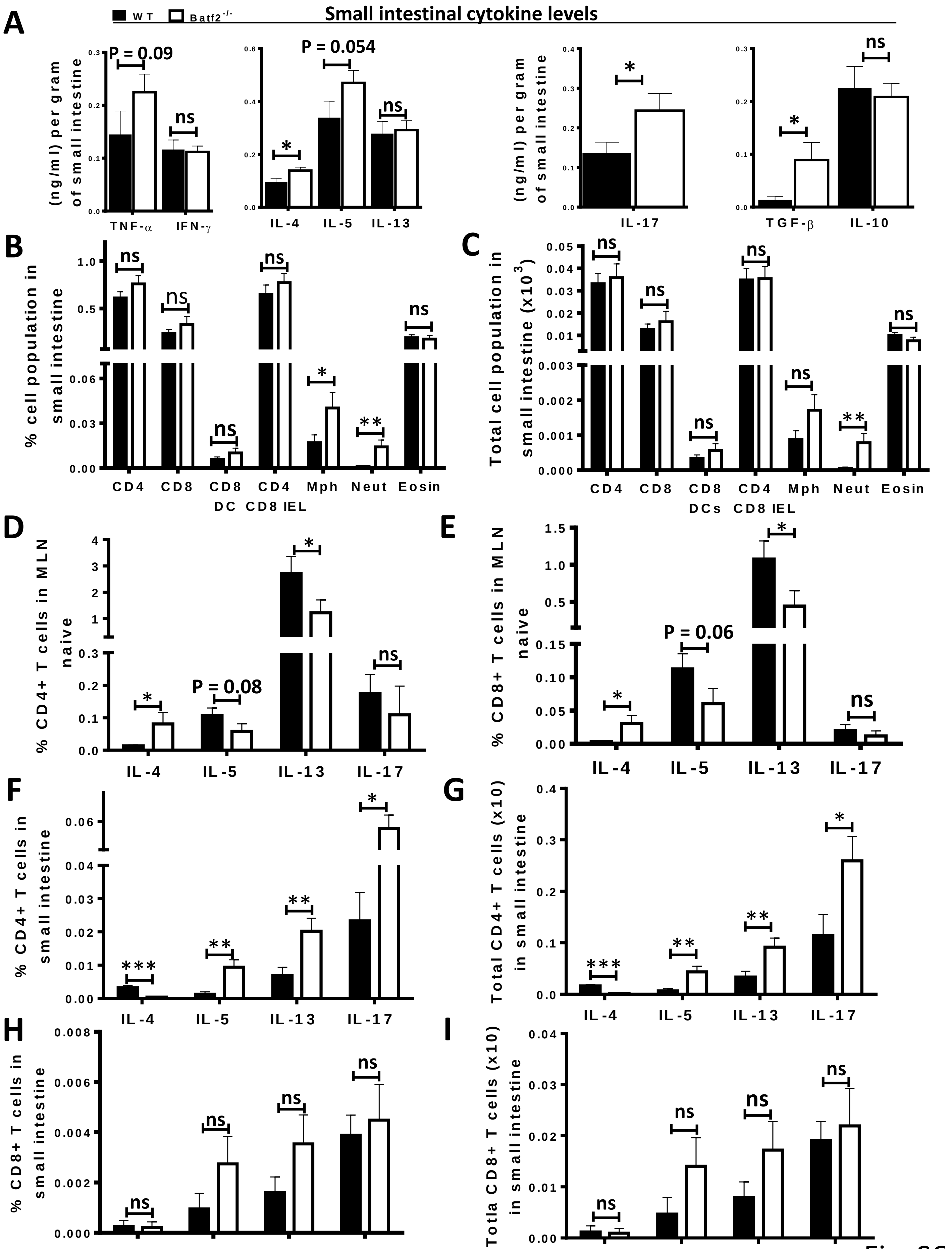
Small intestinal cytokine levels
WT
Batf2-/-
A
(ng/ml) per gram of small intestine
TNF-α
IFN-γ
IL-4
IL-5
IL-13
IL-17
TGF-β
IL-10
P = 0.09
P = 0.054
B
% cell population in small intestine
C
Total cell population in small intestine (x10^3)
CD4
CD8
CD8 DC
CD4 CD8 IEL
Mph
Neut
Eosin
D
% CD4+ T cells in MLN naive
P = 0.08
E
% CD8+ T cells in MLN naive
P = 0.06
F
% CD4+ T cells in small intestine
G
Total CD4+ T cells (x10) in small intestine
H
% CD8+ T cells in small intestine
I
Totla CD8+ T cells (x10) in small intestine

Fig. S6

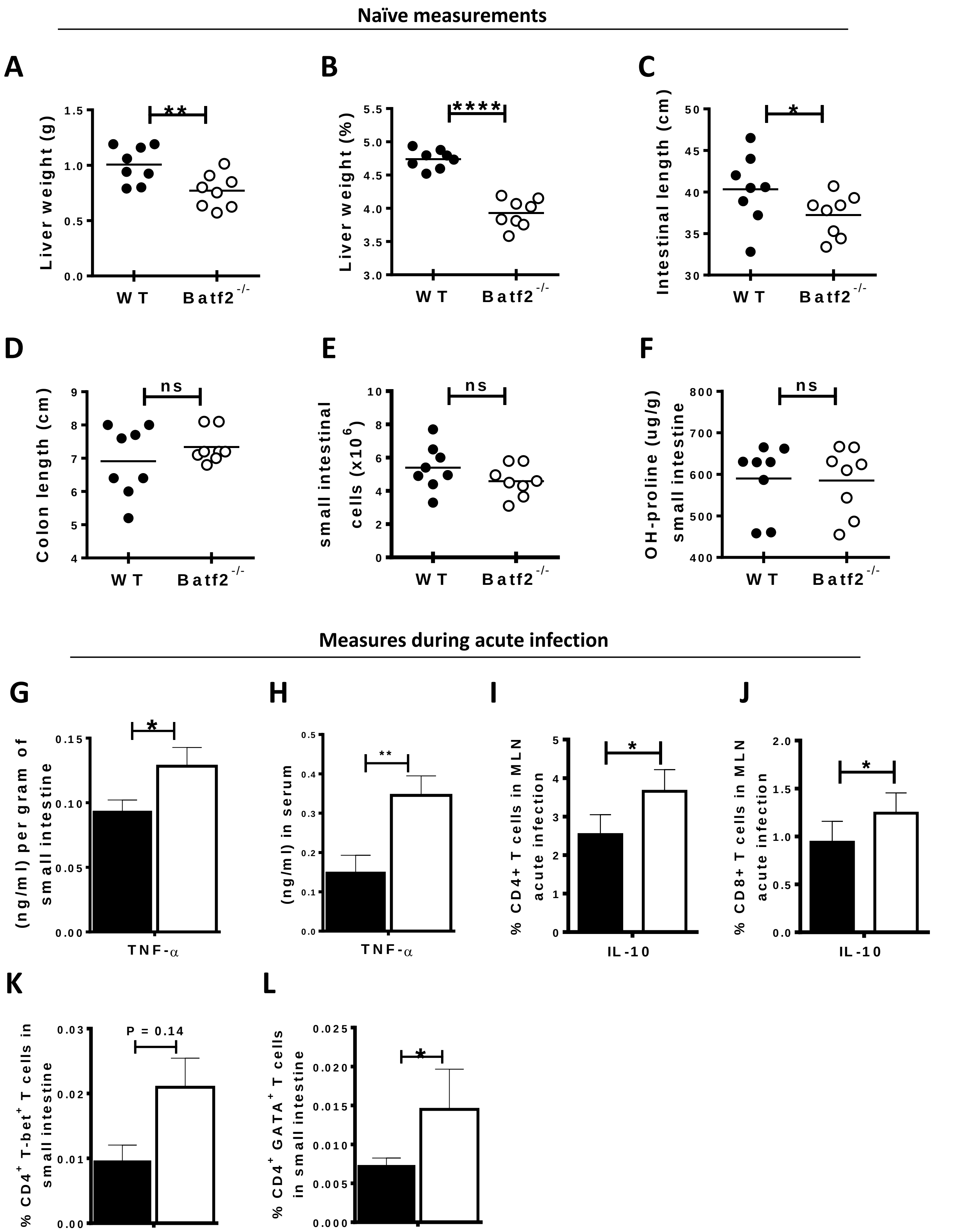
Naïve measurements
A
Liver weight (g)
**
WT
Batf2 -/-
B
Liver weight (%)
****
WT
Batf2 -/-
C
Intestinal length (cm)
*
WT
Batf2 -/-
D
Colon length (cm)
ns
WT
Batf2 -/-
E
small intestinal cells (x10 6)
ns
WT
Batf2 -/-
F
OH-proline (ug/g) small intestine
ns
WT
Batf2 -/-
Measures during acute infection
G
(ng/ml) per gram of small intestine
*
TNF-α
H
(ng/ml) in serum
**
TNF-α
I
% CD4+ T cells in MLN acute infection
*
IL-10
J
% CD8+ T cells in MLN acute infection
*
IL-10
K
% CD4+ T-bet+ T cells in small intestine
P = 0.14
L
% CD4+ GATA+ T cells in small intestine
*

Fig. S7
